# Remarkable Alkene-to-Alkene and Alkene-to-Alkyne Transfer Reactions of Selenium Dibromide and PhSeBr. Stereoselective Addition of Selenium Dihalides to Cycloalkenes

**DOI:** 10.3390/molecules25010194

**Published:** 2020-01-03

**Authors:** Vladimir A. Potapov, Maxim V. Musalov, Evgeny O. Kurkutov, Vladimir A. Yakimov, Alfiya G. Khabibulina, Maria V. Musalova, Svetlana V. Amosova, Tatyana N. Borodina, Alexander I. Albanov

**Affiliations:** A. E. Favorsky Irkutsk Institute of Chemistry, Siberian Division of the Russian Academy of Sciences, 1 Favorsky Str., Irkutsk 664033, Russia; musalov_maxim@irioch.irk.ru (M.V.M.); kurkutov@irioch.irk.ru (E.O.K.); yakimov@irioch.irk.ru (V.A.Y.); almah@irioch.irk.ru (A.G.K.); musalova@irioch.irk.ru (M.V.M.); amosova@irioch.irk.ru (S.V.A.); borodina@irioch.irk.ru (T.N.B.); albanov@irioch.irk.ru (A.I.A.)

**Keywords:** 1-alkenes, alkynes, cycloalkenes, 2-bromoethyl selenides, 2-bromovinyl selenides, phenylselenenyl bromide, selenium dibromide, selenium dihalides, transfer reactions

## Abstract

The original goal of this research was to study stereochemistry of selenium dihalides addition to cycloalkenes and properties of obtained products. Remarkable alkene-to-alkene and alkene-to-alkyne transfer reactions of selenium dibromide and PhSeBr were discovered during this research. The adducts of selenium dibromide with alkenes or cycloalkenes easily exchange SeBr_2_ with other unsaturated compounds, including acetylenes, at room temperature, in acetonitrile. Similar alkene-to-alkene and alkene-to-alkyne transfer reactions of the PhSeBr adducts with alkenes or cycloalkenes take place. The supposed reaction pathway includes the selenium group transfer from seleniranium species to alkenes or alkynes. It was found that the efficient SeBr_2_ and PhSeBr transfer reagents are Se(CH_2_CH_2_Br)_2_ and PhSeCH_2_CH_2_Br, which liberate ethylene, leading to a shift in equilibrium. The regioselective and stereoselective synthesis of bis(*E*-2-bromovinyl) selenides and unsymmetrical *E-*2-bromovinyl selenides was developed based on the SeBr_2_ and PhSeBr transfer reactions which proceeded with higher selectivity compared to analogous addition reactions of SeBr_2_ and PhSeBr to alkynes under the same conditions.

## 1. Introduction 

Selenium is an essential trace element nutrient that functions as cofactor for glutathione peroxidase and certain forms of thioredoxin reductase in humans [1,2,3,4]. Organoselenium compounds exhibit various biological activities, including antitumor, antibacterial, antifungal, anti-inflammatory, and glutathione peroxidase-like actions [1,2,3,4,5,6,7,8,9]. Selenium-containing reagents and organoselenium compounds play an important role in modern organic synthesis [7,8,9,10,11,12,13,14]. Application of novel selenium-containing reagents which allow carrying out regioselective and stereoselective introduction of the selenium atom into organic molecules is an important function.

Efficient electrophilic selenium-containing reagents, selenium dichloride and dibromide, were first introduced in synthesis of organoselenium compounds in 2003 [15,16]. It has been demonstrated that selenium dichloride [17] and dibromide [15] (generated in situ from elemental selenium and sulfuryl chloride or bromine) can be successfully used for selective introduction of the selenium atom into organic molecules [15,16]. Since then, novel chemistry of selenium dihalides has been intensively developed [18,19,20,21,22,23,24,25,26,27,28,29,30,31,32,33,34,35,36,37,38,39,40,41,42,43,44,45,46,47,48,49,50,51,52,53,54,55].

The addition of selenium dichloride and dibromide to double bonds was studied in the reactions with divinyl sulfide [29,30,31], divinyl selenide [32,33,34,35,36], and divinyl sulfone [37,38,39,40,41], affording novel heterocyclic compounds. The transannular addition of selenium dihalides to *cis*,*cis*-cycloocta-1,5-diene gave 2,6-dihalo-9-selenabicyclo[3.3.1]nonanes in high yields [42,43,44]. The reactions of selenium dichloride with allyl and propargyl phenyl ethers afforded annulated products in high yields [45,46,47].

**Scheme 1:**
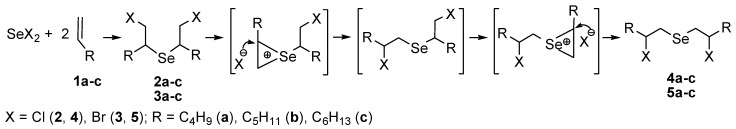
The reactions of selenium dihalides with 1-alkenes.

Recently we studied the addition of selenium halides to 1-alkenes **1a**–**1c** [48,49]. It has been found that the reactions led to anti-Markovnikov adducts, bis(1-haloalk-2-yl) selenides **2a**–**2c** and **3a**–**3c** (kinetic products), which underwent rearrangement to thermodynamically stable Markovnikov adducts, bis(2-haloalkyl) selenides **4a**–**4c** and **5a**–**5c** (Scheme 1). The rearrangement was supposed to proceed via intermediate seleniranium species.

Stereoselectivity of the addition of selenium halides to unsaturated compounds has been basically studied for acetylenes [45,50,51,52,53,54,55,56]. The reactions of selenium dichloride and dibromide with acetylene occurred stereoselectively as *anti*-addition affording (*E,E*)-bis(2-halovinyl) selenides in high yields [50]. The addition of selenium dihalides to mono-substituted acetylenes, as a rule, proceeded in a regioselective and stereoselective mode, giving anti-Markovnikov products with (*E*)-stereochemistry [45,51,52].

In spite of sufficient progress in application of selenium dihalides in synthesis of organoselenium compounds [18,19,20,21,22,23,24,25,26,27,28,29,30,31,32,33,34,35,36,37,38,39,40,41,42,43,44,45,46,47,48,49,50,51,52,53,54,55], the stereochemistry of the addition of these novel electrophilic reagents to the double bond was not examined and required careful studying. Besides, the reactions of selenium dichloride and dibromide with cycloalkenes were not described in the literature. However, studying these reactions may make it possible to determine *syn* or *anti* process takes place on the addition of selenium dihalides to the double bond. We endeavored to study these reactions and stereochemistry of the addition.

## 2. Results and Discussion

**Scheme 2:**
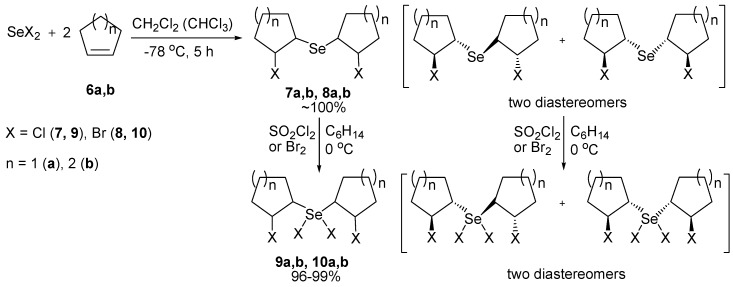
Synthesis of compounds **7a**,**b** and **8a**,**b** and halogenated products **9a**,**b** and **10a**,**b**.

We found that reactions of selenium dihalides with cycloalkenes **6a**,**b** proceeded stereoselectively as *anti*-addition giving hitherto unknown *trans,trans*-bis(2-halocycloalkyl) selenides **7a**,**b** and **8a**,**b** in quantitative yields (Scheme 2).

Favorable conditions for efficient chemoselective and stereoselective reaction consist in addition of selenium dichloride or dibromide to a solution of cycloalkenes **6a**,**b** at −78 °C in methylene chloride or chloroform. These reactions can be carried out at room temperature; however, the selectivity was decreased in this case, and the formation of some by-products in 2–5% yields was observed.

The reliable evidence of the *anti*-addition could be obtained by X-ray studying the adducts of selenium dihalides with cycloalkenes. However, the selenides **7a**,**b** and **8a**,**b** are liquid substances. In order to obtain crystals suitable for X-ray analysis, the halogenation of selenides **7a**,**b** and **8a**,**b**, with bromine and sulfuryl chloride, was carried out. We found that the halogenation reaction occurred efficiently in hexane at 0 °C. Under these conditions, the reaction was accompanied by precipitation of the target products, which can be easily isolated.

**Figure 1:**
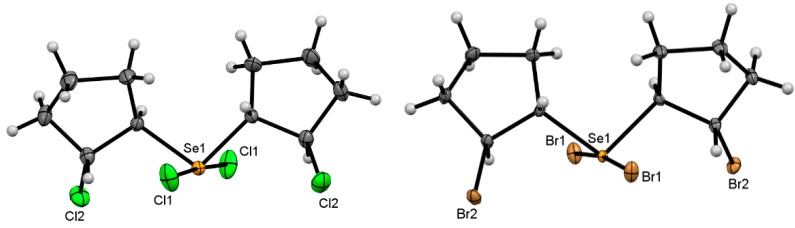
ORTEP molecular structure of selanes **9a** and **10a** (50% thermal ellipsoid probability).

Using this method, we obtained *trans,trans*-dihalo[bis(2-halocycloalkyl)]-λ^4^-selanes **9a**,**b** and **10a**,**b** in 96–99% yields (Scheme 2). The materials suitable for single-crystal X-ray diffraction were obtained from selanes **9a** and **10a**. The X-ray analysis of selanes **9a** and **10a** exhibited *trans,trans*-configuration (Figure 1). The structural assignment of *trans,trans*-configuration of selenides **7a**,**b** and **8a**,**b** was also proved by NMR spectroscopy, including NOESY experiments (see Supplementary Materials), which indicated *trans*-disposition of the protons in the group SeCH-CHCl (no cross-peaks between these protons were observed in ^1^H 2D NOESY spectra). Each of compounds **7a**,**b**, **8a**,**b**, **9a**,**b**, and **10a**,**b** has *trans,trans*-configuration and consist of two diastereomers (Scheme 2), which manifest in the NMR spectra. In the ^13^C-NMR spectra of these compounds, every carbon atom appears as two close signals which correspond to two diastereomers. 

**Scheme 3:**
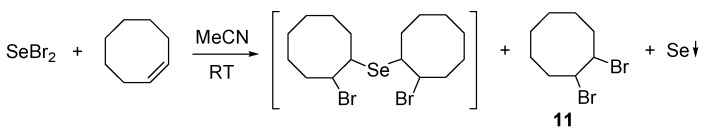
The reaction of selenium dibromide with cyclooctene.

We failed to obtain pure products from the reaction of selenium dihalides and cyclooctene. We supposed that equilibrium may take place between starting compounds and the product in the reaction of selenium dibromide with cyclooctene or other alkenes. The reaction with cyclooctene was carried out in chloroform or methylene chloride, using a 1:2 ratio of the reagents, at room temperature. However, some amount of cyclooctene always remained in the reaction mixture, and the reaction was not accomplished to the end. Adding acetonitrile to the reaction mixture increased conversion and led to halogenation of the double bond, accompanied by the elemental selenium precipitation (Scheme 3). The selenium precipitation led to the shift of the equilibrium, and the formation of 1,2-dibromocyclooctane (**11**) (~40% yield) was observed. It was noted in [57] that the adduct of PhSeCl with cyclooctene was found to be relatively unstable and suffered decomposition over the course of several hours at room temperature.

The anchimeric assistance effect, also known as neighboring-group participation, is usually considered mainly as a factor accelerating the rate of nucleophilic substitution reaction. In the present study, we are facing the new property of this effect, consisting in discovery of remarkable selenium dibromide and organylselenenylbromide transfer reactions.

**Scheme 4:**
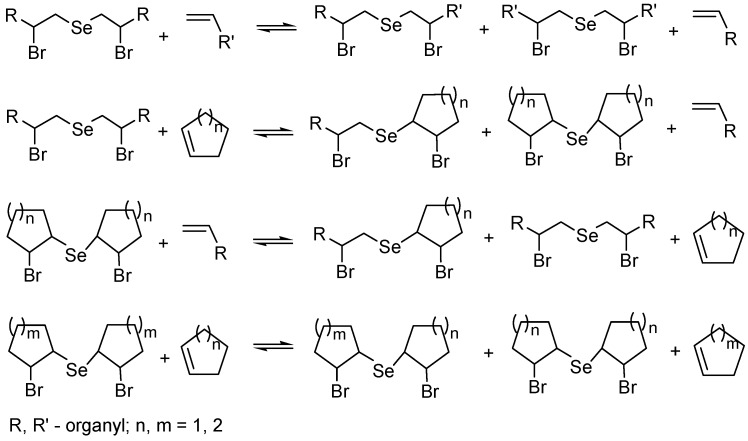
The alkene-to-alkene transfer reactions of selenium dibromide.

Mixing compounds **5a**–**5c** or **8a**,**b** (or their solutions) with alkene or alkyne leads to the formation of new adducts of selenium dibromide. Possible options of the alkene-to-alkene transfer reactions of selenium dibromide are presented in the Scheme 4.

The selenides **5a**–**5c** and **8a**,**b** are believed to exist in equilibrium with seleniranium cations and the transfer reactions proceed via these intermediate species. The driving force for the generation of seleniranium cations is high anchimeric assistance effect of the selenium atom. It was found that this effect is more than one order of magnitude greater than the effect of the sulfur atom [42]. The anchimeric assistance effects of selenium and sulfur atoms have been quantitatively estimated based on the determination of the absolute and relative rates of nucleophilic substitution of chlorine in 2,6-dichloro-9-selena- and -thiabicyclo[3.3.1]nonanes obtained by the transannular addition of selenium or sulfur dichloride to *cis*,*cis*-1,5-cyclooctadiene [42].

**Scheme 5:**
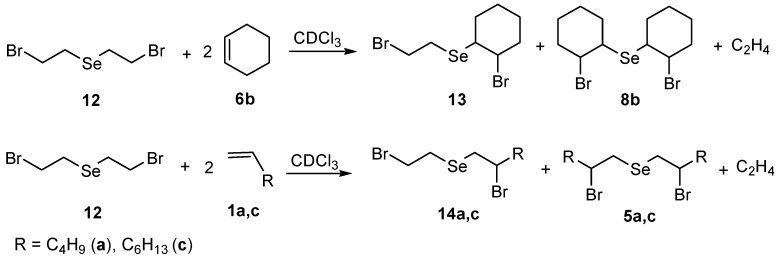
The ^1^H-NMR monitoring of the alkene-to-alkene transfer reactions of SeBr_2_.

We found that a useful reagent to carry out the selenium dibromide transfer reaction is bis(2-bromoethyl) selenide (**12**). The reactions of selenide **12** with 1-alkenes **1a**,**c** or cyclohexene (a 1:2 molar ratio of selenide **12** and the alkene) were monitored by NMR spectroscopy (Scheme 5). The reagents were mixed in CDCl_3_ solution and closed NMR tubes were left at room temperature. The ^1^H and ^13^C-NMR spectra of the samples were recorded at regular intervals. In the case of cyclohexene, a molar ratio of the compounds **12**:**13**:**8b** was 34:51:15 after 10 days. The appearance of the intensive signal of ethylene (5.36 ppm) is noteworthy. The complete conversion of **12** was observed after ~1 month with the formation of compounds **13** and **8b** in approximately equimolar ratio. The reaction of selenide **12** with 1-hexene was faster. The molar ratio of the compounds **12**:**14a**:**5a** was 18:50:32 after four days, and the complete conversion of **12** was observed after two weeks.

**Scheme 6:**
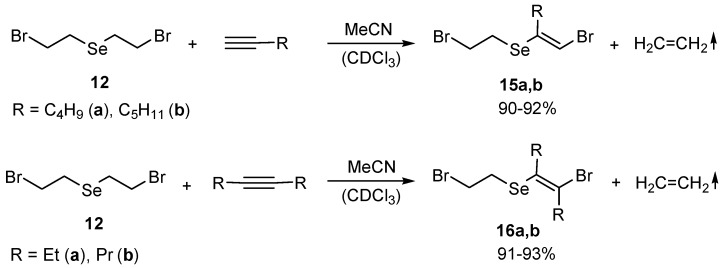
Synthesis of unsymmetrical selenides **15a**,**b** and **16a**,**b**.

Alkynes were involved in the selenium dibromide transfer reactions. The reactions of selenide **12** with 1-hexyne and 3-hexyne (a 1:2 molar ratio of selenide **12** and the alkyne) were also monitored by NMR spectroscopy at room temperature, using CDCl_3_ solutions in closed NMR tubes (Scheme 6). The reactions led to unsymmetrical selenides **15a**,**b** and **16a**,**b**. The complete conversion of selenide **12** was observed after ~15 days in the reaction with 3-hexyne (~90% yield of selenide **16a**). For the same period of time, the conversion of compound **12** was 60% (~55% yield of selenide **15a**) in the reaction with 1-hexyne.

We found that the transfer reactions are considerably accelerated by using acetonitrile as a solvent. Acetonitrile is a polar aprotic solvent with a high dielectric constant (~38), exhibiting the ability to accelerate ionic reactions. The reactions of selenide **12** with terminal (1-hexyne and 1-heptyne) and internal (3-hexyne and 4-octyne) alkynes were carried out in acetonitrile in closed flasks, using a 1:3 molar ratio of selenide **12** and the alkyne (Scheme 6). The complete conversion of selenide **12** and the formation of products **16a**,**b** in 91–93% yield were observed in the reactions with internal alkynes (3-hexyne or 4-octyne) after overnight stirring (14 h) at room temperature. In the case of terminal alkynes (1-hexyne and 1-heptyne), the reactions gave compound **15a**,**b** in 90–92% yield after 30 h of stirring at room temperature.

**Scheme 7:**
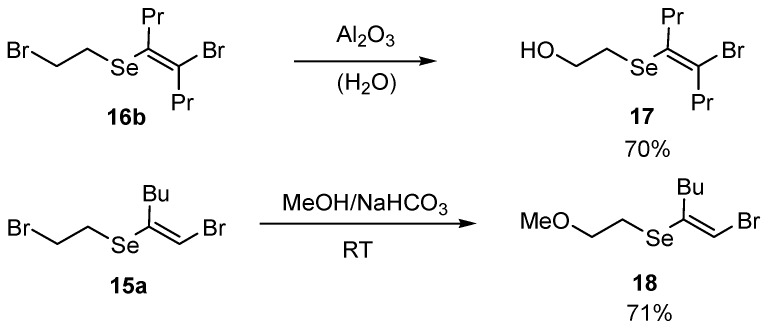
Synthesis of compounds **17** and **18**.

With the goal to purify the product, selenide **16b** was subjected to column chromatography (Al_2_O_3_, hexane → hexane/chloroform 4:1). However, instead of compound **16b**, hydroxyl derivative **17** was isolated in 70% yield (Scheme 7). Obviously, the bromine atom in compound **16b** was substituted by the hydroxyl group due to traces of moisture on the alumina. It is worth noting that the bromine atom in the 2-bromoethylselenide group is very reactive with respect to nucleophilic substitution due to high anchimeric assistance effect of the selenium atom [42].

Taking into account that selenides containing 2-bromoethyl moiety may decompose on purification by column chromatography, the bromine atom was substituted by methoxy group in compound **15a** by reaction with methanol in the presence of NaHCO_3_ at room temperature (Scheme 7). The target product, (1*E*)-1-bromo-2-[(2-methoxyethyl)selanyl]hex-1-ene (**18**), was isolated in 71% yield by column chromatography (Al_2_O_3_, hexane → hexane/chloroform 9:1). The reactions (Scheme 6 and Scheme 7) demonstrate the possibility for selective preparation of unsymmetrical selenides of the type **15a**,**b**–**18** based on selenium dibromide transfer reactions.

The reaction of selenide **12** with 4-octyne (a 1:2 molar ratio) was also monitored by NMR spectroscopy at room temperature, using CD_3_CN solution in a closed NMR tube. The decrease of the content of starting selenide **12** with proportional increasing the contents of compound **16b** and bis[(*E*)-2-bromo-1-propyl-1-pentenyl] selenide (**19b**) was observed. The formation of symmetrical selenide **19b** occurred by the reaction of compound **16b** with 4-octyne. After 145 h, the molar ratio of the compounds **12**:**16b**:**19b** was 34:51:15 (see Supplementary Materials).

**Figure 2:**
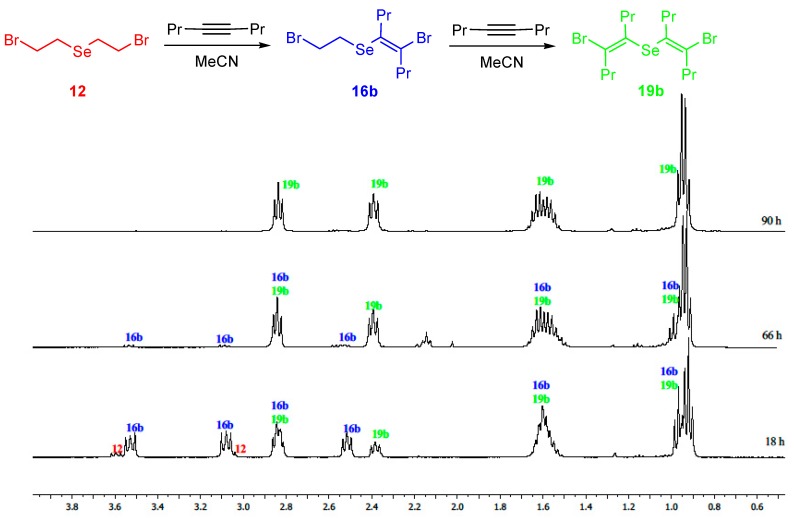
The ^1^H-NMR monitoring of the **16b** and **19b** formation from selenide **12** in MeCN.

When the reactions were carried out in open-air flasks, there was a better possibility for ethylene evolution compared to closed NMR tubes, and the reactions proceeded faster in the open-air flasks. The use of excess alkyne with respect to bis(2-bromoethyl) selenide was also useful for shifting the equilibrium. When the reaction of selenide **12** with 4-octyne (a 1:3.3 molar ratio) was carried out in a closed flask in acetonitrile at room temperature, pure selenide **19b** was obtained in quantitative yield in 90 h (Figure 2).

**Scheme 8:**
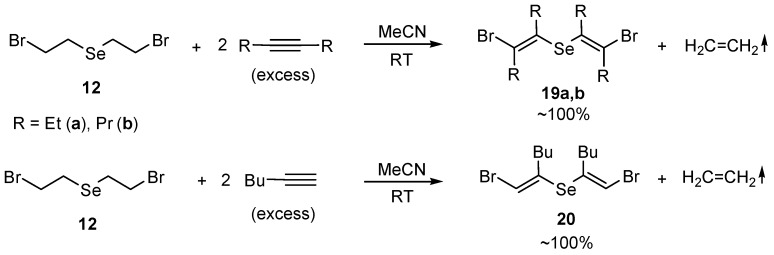
The alkene-to-alkyne transfer reactions of selenium dibromide.

When the flasks were equipped with a tube containing drying agent (CaCl_2_) in order to allow ethylene releasing without moisture access in the flask, the complete conversion of **12** in the reaction with internal alkynes was observed after overnight stirring, and pure divinyl selenides **19a**,**b** were obtained in quantitative yields (Scheme 8).

In the case of 1-hexyne, 40 h stirring at room temperature was required in order to complete the reaction and to produce selenide **20** in a quantitative yield (Scheme 8). The reaction occurred in a regioselective and stereoselective mode affording anti-Markovnikov product of (*E,E*)-stereochemistry.

The fastest version of these reactions consisted in carrying out the process with inert gas bubbling (argon or nitrogen) into the mixture, in order to remove ethylene. The reactions of selenide **12** with excess internal alkynes (3-hexyne and 4-octyne) in acetonitrile were accomplished in 1 h, and the reaction with 1-hexyne was completed in 2 h at room temperature. However, the yields (90–96%) and purity (90–95%) of the crude products **19a**,**b** and **20** were slightly lower compared to those achieved by reactions proceeding in flasks (Scheme 8).

We also studied the direct reactions of selenium dibromide with 3-hexyne and 4-octyne under the same conditions as in Scheme 8. These reactions proceeded in acetonitrile faster than the reactions of compound **12** with 3-hexyne and 4-octyne but less selectively and the formation of some by-products was observed. Changing acetonitrile for methylene chloride as a solvent and decreasing temperature of the reaction to 0 °C allowed to increase the selectivity of the reactions of selenium dibromide with 3-hexyne and 4-octyne and to obtain pure products **19a**,**b** in near quantitative yields.

**Scheme 9:**
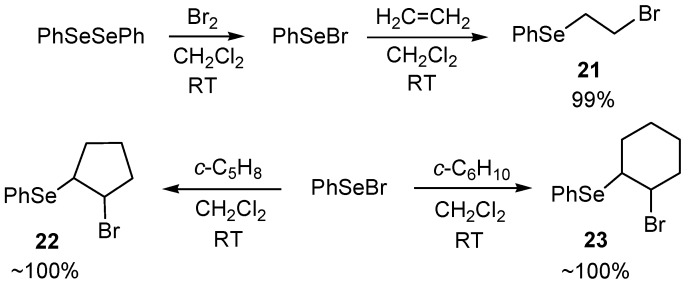
Synthesis of compounds **21**–**23**.

We supposed that similar transfer reactions may also occur with addition products of organylselenenyl bromides to alkenes. We obtained 2-bromoethyl, 2-bromocyclopentyl and 2-bromocyclohexyl phenyl selenides **21**–**23** by addition of phenylselenenyl bromide to ethylene, cyclopentene and cyclohexene (Scheme 9) and studied the PhSeBr transfer reactions of these reagents with some alkenes, cycloalkenes, and alkynes. Indeed, compounds **21**–**23** participated in the phenylselenenyl bromide transfer reactions, which proceeded smoothly at room temperature in acetonitrile. Preliminary results demonstrate that the PhSeBr transfer reactions occur faster compared to the SeBr_2_ transfer reactions with the same alkenes or alkynes under the same conditions.

**Scheme 10:**
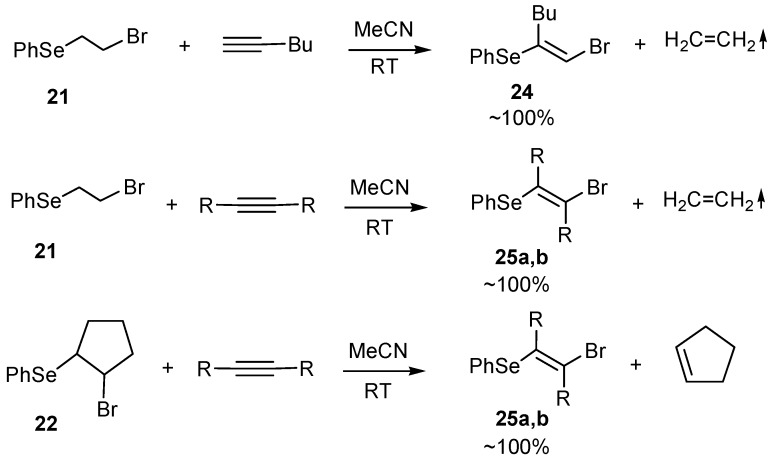
The alkene-to-alkyne transfer reactions of PhSeBr.

The reactions of selenide **21** with excess 1-hexyne, 3-hexyne, and 4-octyne were carried out by stirring the reagents in acetonitrile overnight at room temperature in the flasks equipped with a tube containing a drying agent (CaCl_2_). The reactions proceeded in a stereoselective mode, as *anti*-addition giving products with (*E*)-stereochemistry **24** and **25a**,**b** in quantitative yields (Scheme 10). The regioselective formation of anti-Markovnikov adduct **24** was observed in the reaction with 1-hexyne. The addition of PhSeBr to 1-hexyne under the same conditions in acetonitrile was less selective compared to the PhSeBr transfer reaction giving some by-products (5–7%) along with the target compound **24**.

Surprisingly, adduct **22** can also serve as the efficient PhSeBr transfer reagent liberating cyclopentene (bp 44 °C), which is evaporated during 40 h stirring (Scheme 10).

**Scheme 11:**
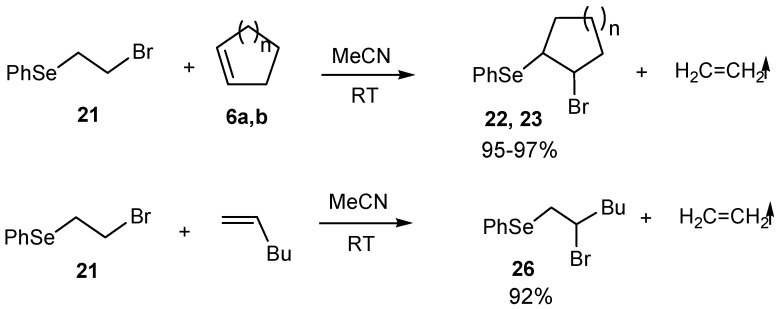
The alkene-to-alkene transfer reactions of PhSeBr.

The alkene-to-alkene PhSeBr transfer reactions also occurred easily at room temperature in acetonitrile. Examples of the alkene-to-alkene PhSeBr transfer (the reactions of selenide **21** with cycloalkenes and 1-hexene) are presented in Scheme 11. The reactions (Scheme 10 and Scheme 11) proceeded very fast (~1 h) when inert gas (argon or nitrogen) was slowly bubbled into the mixture in order to remove ethylene.

**Scheme 12:**
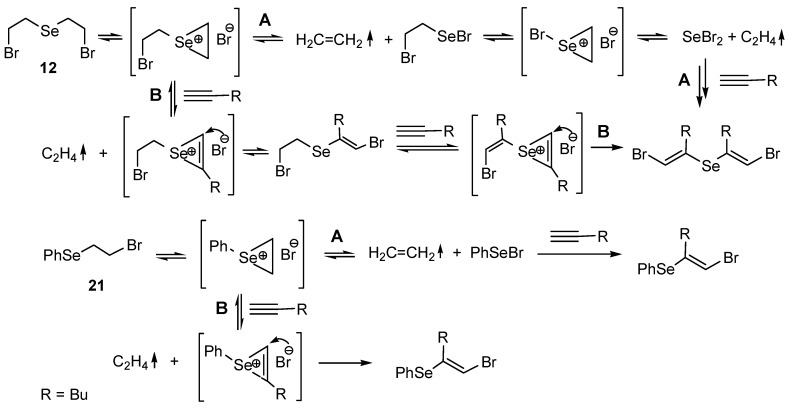
The possible pathways (**A** and **B**) of the alkene-to-alkyne transfer reactions of SeBr_2_ and PhSeBr on the example of reactions of compounds **12** and **21** with 1-hexyne.

Two possible pathways of the SeBr_2_ or PhSeBr transfer reactions can be discussed. If the selenides containing the 2-bromoethyl moiety exist in some equilibrium with the starting compounds, generated SeBr_2_ or PhSeBr may add to other unsaturated compound (pathway **A** is depicted in Scheme 12 on the example of compounds **12**, **21**, and 1-hexyne). Another reaction pathway is based on the assumption that the intermediates involved in the transfer reactions are seleniranium species (pathway **B**, Scheme 12). 

Seleniranium species have been discussed for a long time as reactive intermediates in addition reactions of selenium-centered electrophiles to the double bond [57,58,59,60,61,62,63,64,65,66,67,68,69,70,71,72,73,74]. Some of them have been detected or isolated [57,58,59,71,72,73,74]. Recently, Poleschner and Seppelt isolated and fully characterized series of moderately stable seleniranium and telluriranium salts [73]. The seleniranium ions have been found to play a key role in the process of chirality transfer in asymmetric synthesis reactions studied by Wirth, Santi, Back, and others [60,61,62,63,64,65,66,67]. Wirth and co-workers have postulated facile alkene-to-alkene transfer of selenenium cations in the asymmetric reactions [60,63]. In the reports of Russian scientists [58,59], the enthalpic barrier for seleniranium ion–alkene transfer has been computationally evaluated and found to be considerably lower compared to that of thiiranium ion–alkene transfer. Denmark and co-workers have experimentally observed direct selenium group transfer from phenyl- and butylseleniranium ions to alkenes [57]. The transfer occurred instantaneously at low temperature (−70 °C) in these experiments. We think that the SeBr_2_ and PhSeBr transfer reactions are more likely to follow the pathway **B**, taking into account the ease of the selenium group transfer from seleniranium species to unsaturated bonds. Regiochemistry and stereochemistry of the SeBr_2_ and PhSeBr transfer reactions are determined by the formation of seleniranium species. For example, in the case of monosubstituted acetylene (e.g., 1-hexyne), the attack of bromide anion occurs at the unsubstituted carbon atom of the seleniranium cation leading to anti-Markovnikov products (Scheme 12, pathway **B**). The formation of seleniranium species also determines the reaction course as *anti*-addition.

## 3. Materials and Methods

### 3.1. General Information

X-ray diffraction experiments were carried out on a Bruker D8 Venture Photon 100 CMOS diffractometer with Mo-K_α_ radiation (λ = 0.71073 Å). X-ray crystallographic data for compounds **9a** (CCDC 1965943) and **10a** (CCDC 1502244) are shown in Supplementary Materials. These data can be obtained free of charge via http://www.ccdc.cam.ac.uk/conts/retrieving.html. We recorded ^1^H (400.1 MHz) and ^13^C (100.6 MHz) NMR spectra on a Bruker DPX-400 spectrometer, in 5–10% solution in CDCl_3_. Of note, ^1^H and ^13^C chemical shifts (δ) are reported in parts per million (ppm), relative to the residual solvent peak of CDCl_3_ (δ = 7.27 and 77.00 ppm in ^1^H- and ^13^C-NMR, respectively). Mass spectra were recorded on a Shimadzu GCMS-QP5050A, with electron impact (EI) ionization, at 70 eV. Elemental analysis was performed on a Thermo Flash EA 1112 Elemental Analyzer (USA).

### 3.2. Synthesis of Compounds ***7a**,**b**–**10a**,**b***

*trans,trans-Bis(2-chlorocyclopentyl) selenide* (**7a**). *Typical Procedure*. A solution of selenium dichloride (2.5 mmol) in CH_2_Cl_2_ (20 mL) was added dropwise to a cooled (−78 °C) solution of cyclopentene (0.347 g, 5.1 mmol) in CH_2_Cl_2_ (30 mL). The mixture was stirred at −78 °C for 4 h and 1 h at room temperature. The solvent was removed on a rotary evaporator. The residue was dried in vacuum, giving the product as a yellowish oil. Yield: 0.715 g (quantitative). ^1^H-NMR (400.1 MHz, CDCl_3_): δ 1.70–1.92 (m, 4H, CH_2_), 1.98–2.17 (m, 4H, CH_2_), 2.41–2.55 (m, 4H, CH_2_), 3.58–3.64 (m, 2H, CHSe), 4.41–4.48 (m, 2H, CHCl). ^13^C-NMR (100.6 MHz, CDCl_3_): δ 22.25, 22.30 (CH_2_), 31.27, 31.61 (CH_2_), 34.81, 34.91 (CH_2_), 47.03 (CHSe, ^1^*J*_C-Se_ = 69 Hz), 47.41 (CHSe, ^1^*J*_C-Se_ = 65 Hz), 66.42, 66.60 (CHCl). Anal. Calcd for C_10_H_16_Cl_2_Se: C, 41.98; H, 5.64; Cl, 24.78; Se, 27.60. Found, %: C, 42.25; H, 5.83; Cl, 25.08; Se, 27.29.

*trans,trans-Bis(2-chlorocyclohexyl) selenide* (**7b**) was obtained as a yellowish oil (0.785 g) in quantitative yield from selenium dichloride and cyclohexene under the same conditions as compound **7a**. ^1^H-NMR (400.1 MHz, CDCl_3_): δ 1.44–1.47 (m, 4H, CH_2_), 1.57–1.81 (m, 8H, CH_2_), 2.24–2.38 (m, 4H, CH_2_), 3.25–3.30 (m, 2H, CHSe), 4.23–4.30 (m, 2H, CHCl). ^13^C-NMR (100.6 MHz, CDCl_3_): 22.03 (CH_2_), 22.26 (CH_2_), 23.58 (CH_2_), 23.90 (CH_2_), 30.35 (CH_2_), 33.07 (CH_2_), 45.92 (CHSe, ^1^*J*_C-Se_ = 66 Hz) 46.53 (CHSe, ^1^*J*_C-Se_ = 69 Hz), 63.86 (CHCl), 64.45 (CHCl). Anal. Calcd for C_12_H_20_Cl_2_Se: C, 45.88; H, 6.42; Cl, 22.57; Se, 25.13. Found, %: C, 46.15; H, 6.61; Cl, 22.87; Se, 24.89.

*trans,trans-Bis(2-bromocyclopentyl) selenide* (**8a**) was obtained as a light yellow oil (0.938 g) in quantitative yield from selenium dibromide and cyclopentene under the same conditions as compound **7a**. ^1^H-NMR (400.1 MHz, CDCl_3_): δ 1.76–1.91 (m, 4H, CH_2_), 1.96–2.07 (m, 2H, CH_2_), 2.11–2.18 (m, 2H, CH_2_), 2.43–2.59 (m, 4H, CH_2_), 3.67–3.77 (m, 2H, CHSe), 4.43–4.54 (m, 2H, CHBr). ^13^C-NMR (100.6 MHz, CDCl_3_): δ 22.39 (CH_2_), 22.45 (CH_2_), 31.22 (CH_2_), 31.53 (CH_2_), 35.27 (CH_2_), 35.41 (CH_2_), 47.74 (CHSe, ^1^*J*_CSe_ = 67 Hz) 48.16 (CHSe, ^1^*J*_CSe_ = 64 Hz), 57.18 (CHBr), 57.22 (CHBr). Anal. Calcd for C_10_H_16_Br_2_Se: C, 32.03; H, 4.30; Br, 42.62; Se, 21.08. Found, %: C, 31.85; H, 4.12; Br, 42.98; Se, 20.73.

*trans,trans-Bis(2-bromocyclohexyl) selenide* (**8b**) was obtained as a light yellow oil (1.008 g) in quantitative yield from selenium dibromide and cyclohexene under the same conditions as compound **7a**. ^1^H-NMR (400.1 MHz, CDCl_3_): δ 1.49–1.57 (m, 6H, CH_2_), 1.74–1.79 (m, 4H, CH_2_), 1.89–1.93 (m, 2H, CH_2_), 2.29–2.43 (m, 4H, CH_2_), 3.38–3.41 (m, 2H, CHSe), 4.53–4.58 (m, 2H, CHBr). ^13^C-NMR (100.6 MHz, CDCl_3_): δ 22.41 (CH_2_), 23.36 (CH_2_), 23.57 (CH_2_), 30.37 (CH_2_), 32.90 (CH_2_), 46.65 (CHSe, ^1^*J*_CSe_ = 66 Hz), 47.07 (CHSe, ^1^*J*_CSe_ = 67 Hz), 57.73 (CHBr), 58.06 (CHBr). Anal. Calcd for C_12_H_20_Br_2_Se: C, 35.76; H, 5.00; Br, 39.65; Se, 19.59. Found, %: C, 35.48; H, 4.85; Br, 40.03; Se, 19.82.

*trans,trans-Dichloro[bis(2-chlorocyclopentyl)]-λ^4^-selane* (**9a**)**.** A solution of sulfuryl chloride (0.27 g, 2 mmol) in hexane (10 mL) was added to a cooled to −0 °C solution of selenide **7a** (0.572 g, 2 mmol) in hexane (15 mL) and the mixture was stirred at −0 °C for 8 h and allowed to warm to room temperature. The precipitate was filtered off and dried in vacuum to give the product as a white powder, mp = 114–115 °C. Yield: 0.685 g (96%). The crystals suitable for single-crystal X-ray diffraction were obtained by recrystallization from chloroform. ^1^H-NMR (400.1 MHz, CDCl_3_): δ 1.98–2.03 (m, 6H, CH_2_), 2.38–2.42 (m, 4H, CH_2_), 2.63–2.70 (m, 2H, CH_2_), 4.59–4.68 (m, 2H, CHSe), 4.98–4.99 (m, 2H, CHCl). ^13^C-NMR (100.6 MHz, CDCl_3_): δ 23.20 (CH_2_), 29.49 (CH_2_), 29.65 (CH_2_), 36.54 (CH_2_), 36.71 (CH_2_), 60.29 (CHCl), 60.35 (CHCl), 77.51 (CHSe), 78.07 (CHSe). Anal. Calcd for C_10_H_16_Cl_4_Se: C, 35.64; H, 4.52; Cl, 39.72; Se, 22.12. Found, %: C, 35.36; H, 4.33; Cl, 40.05; Se, 21.81.

*trans,trans-Dichloro[bis(2-chlorocyclohexyl)]-λ^4^-selane* (**9b**) was obtained in 96% yield under the same conditions as compound **9a** as a white powder (0.739 g), mp = 133–135 °C. ^1^H-NMR (400.1 MHz, CDCl_3_): δ 1.39–1.49 (m, 4H, CH_2_), 1.76–1.84 (m, 3H, CH_2_), 1.85–1.93 (m, 3H, CH_2_), 2.04–2.16 (m, 1H, CH_2_), 2.29–2.33 (m, 1H, CH_2_), 2.40–2.44 (m, 2H, CH_2_), 2.47–2.55 (m, 1H, CH_2_), 2.85–2.88 (m, 1H, CH_2_), 4.24–4.36 (m, 2H, CHSe), 4.50–4.63 (m, 2H, CHCl). ^13^C-NMR (100.6 MHz, CDCl_3_): δ 25.22, 25.39 (CH_2_), 25.75, 25.97 (CH_2_), 30.24, 30.41 (CH_2_), 38.23, 38.60 (CH_2_), 58.67 (CHCl), 79.03 (CHSe). Anal. Calcd for C_12_H_20_Cl_4_Se: C, 37.43; H, 5.24; Cl, 36.83; Se, 20.51. Found, %: C, 37.34; H, 5.41; Cl, 37.18; Se, 20.87.

*trans,trans-Dibromo[bis(2-bromocyclopentyl)]-λ^4^-selane* (**10a**)**.** A solution of bromine (0.32 g, 2 mmol) in hexane (10 mL) was added to a cooled to 0 °C solution of selenide 10a (0.75 g, 2 mmol) in hexane (15 mL), and the mixture was stirred at 0 °C for 4 h and allowed to warm to room temperature. The precipitate was filtered off and dried in vacuum, to give the product as a yellow powder, mp = 101–102 °C. Yield: 1.06 g (99%). The crystals suitable for single-crystal X-ray diffraction were obtained by recrystallization from chloroform. ^1^H-NMR (400.1 MHz, CDCl_3_): 2.03–2.06 (m, 4H, CH_2_), 2.21–2.23 (m, 2H, CH_2_), 2.46–2.54 (m, 4H, CH_2_), 2.70–2.73 (m, 2H, CH_2_), 4.65–4.74 (m, 2H, CHSe), 5.07–5.08 (m, 2H, CHBr). ^13^C-NMR (100.6 MHz, CDCl_3_): δ 23.71 (CH_2_), 23.80 (CH_2_), 30.66 (CH_2_), 30.95 (CH_2_), 37.73 (CH_2_), 38.00 (CH_2_), 50.50 (CHBr), 50.53 (CHBr), 75.34 (CHSe), 76.06 (CHSe). Anal. Calcd for C_10_H_16_Br_4_Se: C, 22.46; H, 3.02; Br, 59.76; Se, 14.76. Found, %: C, 22.18; H, 2.87; Br, 60.13; Se, 15.05.

*trans,trans-Dibromo[bis(2-bromocyclohexyl)]-λ^4^-selane* (**10b**) was obtained in 98% yield under the same conditions as compound **10a** as a yellow powder (1.103 g), mp = 95–96 °C. ^1^H-NMR (400.1 MHz, CDCl_3_): δ 1.44–1.57 (m, 4H, CH_2_), 1.75–1.84 (m, 2H, CH_2_), 1.90–1.98 (m, 2H, CH_2_), 2.02–2.11 (m, 2H, CH_2_), 2.17–2.27 (m, 2H, CH_2_), 2.31–2.35 (m, 2H, CH_2_), 2.51–2.58 (m, 2H, CH_2_), 2.66–2.76 (m, 2H, CH_2_), 2.92–2.95 (m, 2H, CH_2_), 4.23–4.38 (m, 2H, CHSe), 4.75–4.87 (m, 2H, CHBr). ^13^C-NMR (100.6 MHz, CDCl_3_): δ 25.00 (CH_2_), 26.10 (CH_2_), 31.84, 31.96 (CH_2_), 39.18, 39.66 (CH_2_), 53.21, 55.37 (CHBr), 72.95, 75.41 (CHSe). Anal. Calcd for C_12_H_20_Br_4_Se: C, 25.61; H, 3.58; Br, 56.78; Se, 14.03. Found, %: C, 25.33; H, 3.39; Br, 57.19; Se, 13.82.

### 3.3. Synthesis of Compounds ***17**–**26***

*(4E)-4-Bromo-5-[(2-hydroxyethyl)selanyl]oct-4-ene* (**17**). A solution of 4-octyne (0.11 g, 1 mmol) in MeCN (1 mL) was added to a solution of selenide **12** (0.1 g, 0.34 mmol) in MeCN (1.5 mL), and the mixture was stirred in a 200 mL round-bottomed closed flask overnight (14 h) at room temperature. The solvent was removed by a rotary evaporator. The residue contained compounds **16b** (0.117 g, 91% yield) and **19b** in a 20:1 molar ratio (the NMR data). The residue was subjected to column chromatography (Al_2_O_3_, hexane → hexane/chloroform 4:1). Instead of compound **16b**, hydroxyl derivative **17** (0.068 g, 70% yield based on compound **16b**) was isolated as a colorless oil. ^1^H-NMR (400 MHz, CDCl_3_): δ 3.78–3.75 (m, 2H), 2.91–2.84 (m, 4H), 2.53–2.49 (m, 2H), 1.65–1.54 (m, 4H), 0.98–0.92 (m, 6H). ^13^C-NMR (100.6 MHz, CDCl_3_): δ: 129.50, 126.83, 61.62, 43.21, 40.32, 30.03, 21.88, 21.36, 13.52, 13.01. MS (EI): *m*/*z* (%) 314 (20) [M^+**·**^], 207 (9), 149 (9), 109 (50), 67 (100), 41 (64). Anal. Calcd. For C_10_H_19_BrOSe (314.12): C, 38.24; H, 6.10; Br, 25.44; Se, 25.14%. Found, %: C, 38.52; H, 5.98; Br, 25.19; Se, 24.87%.

*(1E)-1-Bromo-2-[(2-methoxyethyl)selanyl]hex-1-ene* (**18**). A solution of 1-hexyne (0.082 g, 1 mmol) in MeCN (1 mL) was added to a solution of selenide **12** (0.1 g, 0.34 mmol) in MeCN (1.5 mL), and the mixture was stirred in a 200 mL round-bottomed closed flask for 30 h at room temperature. The solvent was removed by a rotary evaporator. The residue contained compounds **15a** (0.107 g, 90% yield) and unconverted selenide **12** in a 9:1 molar ratio (the NMR data). The residue was dissolved in chloroform (1 mL) and methanol (0.3 mL) and NaHCO_3_ (0.084 g, 1 mmol) were added. The mixture was stirred for 24 h at room temperature and then filtered; solvents were removed by a rotary evaporator. The residue was subjected to column chromatography (Al_2_O_3_, hexane → hexane/chloroform 9:1), giving compound **18** (0.065 g, 71% yield based on compound **15a**) as a colorless oil. ^1^H-NMR (400 MHz, CDCl_3_): δ 6.33 (s, 1H) 3.63–3.59 (m, 2H), 3.38 s, 2.90–2.87 (m, 2H), 2.48–2.44 (m, 2H), 1.55–1.51 (m, 2H), 1.41–1.35 (m, 2H), 0.97–0.93 (m, 3H). ^13^C-NMR (100 MHz, CDCl_3_): δ 134.56, 103.64, 71.71, 58.67, 35.17, 29.81, 25.44, 22.16, 13.93. MS (EI): *m*/*z* (%) = 300 (20) [M^+·^], 242(10), 202 (22), 163 (33), 108 (21), 79 (57), 59 (100). Anal. Calcd. for C_9_H_17_BrOSe (300.09): C, 36.02; H, 5.71; Br, 26.63; Se, 26.31%. Found, %: C, 35.75; H, 5.56; Br, 26.39; Se, 26.11%.

*Bis(E-2-bromo-1-ethyl-1-butenyl) selenide* (**19a**). A solution of 3-hexyne (0.14 g, 1.7 mmol) in MeCN (1 mL) was added to a solution of selenide **12** (0.1 g, 0.34 mmol) in MeCN (1 mL). The mixture was stirred in a 100 mL round-bottomed flask equipped with a tube containing a drying agent (CaCl_2_) overnight (18 h) at room temperature. The solvent was removed by a rotary evaporator, and the residue was dried in vacuum, giving compound **19a** (0.137 g) as a light-yellow oil in quantitative yield. ^1^H-NMR (400 MHz, CDCl_3_): δ 2.85 (q, *J* = 7.3 Hz, 4H), 2.41 (q, *J* = 7.3 Hz, 4H), 1.12 (t, *J* = 7.3 Hz, 6H), 1.07 (t, *J* = 7.3 Hz, 6H). ^13^C NMR (100 MHz, CDCl_3_): δ 129.84 (^1^*J*_C-Se_ = 108.7 Hz), 129.49, 35.06 (^2^*J*_C-Se_ = 10.6 Hz), 32.57, 13.42, 12.00. Anal. Calcd. For C_12_H_20_Br_2_Se (403.06): C, 35.76; H, 5.00; Br, 39.65; Se, 19.59%. Found: C, 36.04; H, 4.87; Br, 39.42; Se, 19.87%.

*Bis(E-2-bromo-1-propyl-1-pentenyl) selenide* (**19b**) was obtained in quantitative yield as a light-yellow oil (0.156 g) from 4-octyne and selenide **12** in acetonitrile under the same conditions as compound **19a**. ^1^H-NMR (400 MHz, CDCl_3_): δ 2.83 (t, *J* = 7.5 Hz, 4H), 2.38 (t, *J* = 7.5 Hz, 4H), 1.64–1.53 (m, 8H), 0.95–0.90 (m, 12H). ^13^C-NMR (100 MHz, CDCl_3_): δ 129.79, 128.38, 43.04, 40.79, 21.89, 21.10, 13.56, 12.95. Anal. Calcd. For C_16_H_28_Br_2_Se (459.16): C, 41.85; H, 6.15; Br, 34.80; Se, 17.20%. Found: C, 42.14; H, 5.98; Br, 35.07; Se, 17.03%.

*Bis[(1E)-1-bromohex-1-en-2-yl] selenide* (**20**)**.** A solution of 1-hexyne (0.137 g, 1.67 mmol) in MeCN (1 mL) was added to a solution of selenide **12** (0.065 g, 0.22 mmol) in MeCN (1 mL). The mixture was stirred in a 100 mL round-bottomed flask equipped with a tube containing a drying agent (CaCl_2_) for 40 h at room temperature. The solvent was removed by a rotary evaporator, and the residue was dried in vacuum, giving compound **20** (0.089 g) as a light-yellow oil in quantitative yield. ^1^H-NMR (400 MHz, CDCl_3_): δ 6.45 (s, 2H), 2.46–2.42 (m, 4H), 1.56–1.48 (m, 4H), 1.41–1.32 (m, 4H), 0.96–0.93 (m, 6H). ^13^C NMR (100 MHz, CDCl_3_): δ 134.97, 107.40, 34.77, 29.68, 22.12, 13.90. Anal. Calcd. for C_12_H_20_Br_2_Se (403.06): C, 35.76; H, 5.00; Br, 39.65; Se, 19.59%. Found, %: C, 35.91; H, 4.89; Br, 39.42; Se, 19.33%.

*2-Bromoethyl phenyl selenide* (**21**). Dry ethylene was bubbled to a flask containing CH_2_Cl_2_ (10 mL) with stirring for 10 min at room temperature. A solution of PhSeBr [(4 mmol), prepared from Ph_2_Se_2_ (0.624 g, 2 mmol) and bromine (0.320 g, 2 mmol) in CH_2_Cl_2_ (15 mL)] was added dropwise to the flask for 30 min with stirring. The ethylene bubbling (~30 mL/min) was continued during the PhSeBr addition and 40 min after the addition. The mixture was stirred additionally for 1 h at room temperature and filtered. The solvent was removed in vacuum, giving selenide **21** (1.046 g, 99% yield) as a light-yellow oil. ^1^H-NMR (400 MHz, CDCl_3_): δ 7.56–7.53 (m, 2H), 7.32–7.30 (m, 3H), 3.59–3.55 (m, 2H), 3.30–3.26 (m, 2H). ^13^C-NMR (100 MHz, CDCl_3_): δ 133.25, 129.20, 128.11, 127,57, 30.58, 28.55. Anal. Calcd. for C_8_H_9_BrSe (264.02): C, 36.39; H, 3.44; Br, 30.26; Se, 29.91%.

*2-Bromocyclopentyl phenyl selenide* (**22**). A solution of PhSeBr [(2 mmol), prepared from Ph_2_Se_2_ (0.312 g, 1 mmol) and bromine (0.16 g, 1 mmol) in CH_2_Cl_2_ (10 mL)] was added dropwise to a solution of cyclopentene (0.15 g, 2.2 mmol) in CH_2_Cl_2_ (10 mL), at such a rate that discoloration of the reaction mixture occurred after each drop. The mixture was stirred for 1 h at room temperature, and the solvent was removed on a rotary evaporator. The residue was dried in vacuum, giving the product as a light-yellow oil. Yield: 0.608 g (quantitative). ^1^H-NMR (400 MHz, CDCl_3_): δ 1.80–1.89 (m, 2H, CH_2_), 2.02–2.17 (m, 2H, CH_2_), 2.51–2.59 (m, 2H, CH_2_), 4.08–4.10 (m, 1H, CHSe), 4.50–4.51 (m, 1H, CHBr), 7.32–7.33 (m, 3H, CH_Ar_), 7.57 (m, 2H, CH_Ar_). ^13^C-NMR (100.6 MHz, CDCl_3_): δ 22.30 (CH_2_), 29.93 (CH_2_), 34.80 (CH_2_), 50.40 (CHSe, ^1^*J*_C-Se_ = 65 Hz), 58.58 (CHBr), 127.49 (CH_Ar_), 129.19 (CH_Ar_), 131.36 (CH_Ar_), 133.40 (CH_Ar_). Anal. Calcd for C_11_H_13_BrSe: C, 43.45; H, 4.31; Br, 26.28; Se, 25.97. Found, %: C, 43.67; H, 4.49; Br, 26.48; Se, 26.21.

*2-Bromocyclohexyl phenyl selenide* (**23**) was obtained in quantitative yield as a light-yellow oil (0.636 g) from PhSeBr and cyclohexene in CH_2_Cl_2_ under the same conditions as compound **22.** ^1^H-NMR (400.1 MHz, CDCl_3_): δ 1.52–1.67 (m, 3H, CH_2_), 1.81–1.85 (m, 2H, CH_2_), 1.96–1.97 (m, 1H, CH_2_), 2.39–2.47 (m, 2H, CH_2_), 3.73–3.74 (m, 1H, CHSe), 4.55 (m, 1H, CHBr), 7.27–7.33 (m, 3H, CH_Ar_), 7.61–7.63 (m, 2H, CH_Ar_). ^13^C-NMR (100.6 MHz, CDCl_3_): δ 22.32 (CH_2_), 22.38 (CH_2_), 23.14 (CH_2_), 29.05 (CH_2_), 29.08 (CH_2_), 29.09 (CH_2_), 32.30 (CH_2_), 32.33 (CH_2_), 48.90 (CHSe, ^1^*J*_C,Se_ 64 Hz), 56.57 (CHBr), 127.57 (CH_Ar_), 128.92 (CH_Ar_), 131.16 (CH_Ar_), 134.35 (CH_Ar_). Anal. Calcd for C_12_H_15_BrSe: C, 45.31; H, 4.75; Br, 25.12; Se, 24.82. Found, %: C, 43.05; H, 4.93; Br, 24.86; Se, 25.09.

*(1E)-1-Bromohex-1-en-2-yl phenyl selenide* (**24**)**.** A solution of 1-hexyne (0.091 g, 1.1 mmol) in MeCN (1 mL) was added to a solution of selenide **21** (0.073 g, 0.28 mmol) in MeCN (1 mL). The mixture was stirred in a 100 mL round-bottomed flask equipped with a tube containing a drying agent (CaCl_2_) for 30 h at room temperature. The solvent was removed by a rotary evaporator, and the residue was dried in vacuum, giving compound **24** (0.089 g) as a light-yellow oil in quantitative yield. ^1^H-NMR (400 MHz, CDCl_3_): δ 7.52–7.50 (m, 2H), 7.32–7.27 (m, 3H), 6.39 (s, 1H), 2.45–2.41 (m, 2H), 1.58–1.51 (m, 2H), 1.37–1.30 (m, 2H), 0.93–0.89 (m, 3H). ^13^C-NMR (100 MHz, CDCl_3_): δ 136.59, 133.58, 129.38, 129.02, 127.91, 105.98, 34.69, 29.78, 22.08, 13.88. Anal. Calcd. for C_12_H_15_BrSe (318.11): C, 45.31; H, 4.75; Br, 25.12; Se, 24.82%. Found, %: C, 45.17; H, 4.58; Br, 24.85; Se, 25.09%.

*(3E)-4-Bromohex-3-en-3-yl phenyl selenide* (**25a**). A solution of 3-hexyne (0.091 g, 1.1 mmol) in MeCN (1 mL) was added to a solution of selenide **21** (0.1 g, 0.38 mmol) in MeCN (1 mL). The mixture was stirred in a 100 mL round-bottomed flask equipped with a tube containing a drying agent (CaCl_2_) overnight (14 h) at room temperature. The solvent was removed by a rotary evaporator, and the residue was dried in vacuum, giving compound **25a** (0.12 g) as a light-yellow oil in quantitative yield. ^1^H-NMR (400 MHz, CDCl_3_): δ 7.43–7.41 (m, 2H), 7.29–7.27 (m, 3H), 2.95–2.89 (m, 2H), 2.50–2.45 (m, 2H), 1.16–1.12 (m, 3H), 1.07–1.03 (m, 3H). ^13^C NMR (100 MHz, CDCl_3_): δ 131.99, 130.64, 130.62, 129.83, 129.23, 127.08, 35.37, 32.69, 13.48, 12.34. Anal. Calcd. for C_12_H_15_BrSe (318.11): C, 45.31; H, 4.75; Br, 25.12; Se, 24.82%. Found, %: C, 45.58; H, 4.61; Br, 25.31; Se, 25.11%.

*(4E)-5-Bromooct-4-en-4-yl phenyl selenide* (**25b**) was obtained in quantitative yield as a light-yellow oil (0.131 g) from 4-octyne and selenide **21** in acetonitrile under the same conditions as compound **25a**. ^1^H-NMR (400 MHz, CDCl_3_): δ 7.43–7.41 (m, 2H), 7.29–7.27 (m, 3H), 2.93–2.89 (m, 2H), 2.45–2.41 (m, 2H), 1.67–1.53 (m, 4H), 0.96–0.92 (m, 3H), 0.90–0.86 (m, 3H). ^13^C-NMR (100 MHz, CDCl_3_): δ 132.11, 130.59, 129.50, 129.20, 129.15, 127.07, 43.30, 40.66, 21.86, 21.28, 13.44, 13.00. Anal. Calcd. for C_14_H_19_BrSe (346.16): C, 48.58; H, 5.53; Br, 23.08; Se, 22.81%. Found: C, 48.34; H, 5.38; Br, 22.81; Se, 23.04%.

*The experiment under Ar.* A solution of 4-octyne (0.11 g, 1 mmol) in MeCN (1 mL) was added to a solution of selenide **21** (0.066 g, 0.25 mmol) in MeCN (1 mL), and argon was bubbled into the mixture for 1 h. The solvent was removed by a rotary evaporator and the residue was dried in vacuum, giving compound **25b** (0.084 g, 97% yield) as a light-yellow oil (~95% purity).

*2-Bromohexyl phenyl selenide* (**26**). A solution of 1-hexene (0.185 g, 2.2 mmol) in MeCN (1 mL) was added to a solution of selenide **21** (0.074 g, 0.28 mmol) in MeCN (1.5 mL), and the mixture was stirred in a 200 mL round-bottomed closed flask for 24 h at room temperature. The solvent was removed by a rotary evaporator. The residue (0.089 g) was dried in vacuum, giving the product **25** (~92% purity) as a light-yellow oil. Yield: 92%. ^1^H-NMR (400 MHz, CDCl_3_): δ 7.55–7.53 (m, 2H), 7.31–7.27 (m, 3H), 4.21–4.14 (m, 1H), 3.58–3.53 (m, 1H), 3.35–3.29 (m, 1H), 2.16–2.08 (m, 1H), 1.84–1.75 (m, 1H), 1.54–1.27(m, 4H), 0.93–0.89 (m, 3H). ^13^C-NMR (100 MHz, CDCl_3_): δ 133.21, 129.26, 128.10, 127.49, 55.34, 36.97, 36.35, 29.26, 21.94, 13.87.

## 4. Conclusions

Remarkable alkene-to-alkene and alkene-to-alkyne transfer reactions of selenium dibromide and PhSeBr have been discovered. The compounds containing the 2-bromoethylselanyl moiety easily exchange SeBr_2_ or RSeBr with alkenes, cycloalkenes, and alkynes at room temperature. The efficient SeBr_2_ and PhSeBr transfer reagents are bis(2-bromoethyl) selenide and 2-bromoethyl phenyl selenide, which liberate ethylene, leading to a shift in equilibrium. The favorable conditions include the use of acetonitrile as a solvent and removing the formed alkene (e.g., ethylene or cyclopentene). The regioselective and stereoselective synthesis of *E*-2-bromovinyl selenides **15**–**20**, **24**, and **25a**,**b** in up to quantitative yields was developed based on the SeBr_2_ and PhSeBr transfer reactions, which proceeded with higher selectivity compared to analogous addition reactions of SeBr_2_ and PhSeBr to alkynes under the same conditions. High selectivity, quantitative yields, mild-reaction conditions, and very simple work-up procedures are important features of this approach.

The reactions of selenium dihalides with cycloalkenes proceed stereoselectively as *anti*-addition, affording hitherto unknown *trans,trans*-bis(2-halocycloalkyl) selenides **7a**,**b** and **8a**,**b** in quantitative yields (Scheme 2). The reliable evidence of the *anti*-addition has been obtained by X-ray analysis of dihalo[bis(2-halocycloalkyl)]-λ^4^-selanes **9a** and **10a**, as well as by NMR spectroscopy studies of selenides **7a**,**b** and **8a**,**b**.

## Supplementary Materials

The following are available online, synthesis of compounds **12**–**16a**,**b** and monitoring data, Figure S1, examples of ^1^H- and ^13^C-NMR spectra of the obtained compounds, X-ray crystallographic data of compounds **9a** and **10a**.
