# Supplementary material for: Remarkable Alkene-to-Alkene and Alkene-to-Alkyne Transfer Reactions of Selenium Dibromide and PhSeBr. Stereoselective Addition of Selenium Dihalides to Cycloalkenes
Source: Molecules. 2020 Jan 3;25(1):194. doi: 10.3390/molecules25010194 (PMC6983007; doi:10.3390/molecules25010194)

checkCIF/PLATON report

You have not supplied any structure factors. As a result the full set of tests cannot be run.

THIS REPORT IS FOR GUIDANCE ONLY. IF USED AS PART OF A REVIEW PROCEDURE FOR PUBLICATION, IT SHOULD NOT REPLACE THE EXPERTISE OF AN EXPERIENCED CRYSTALLOGRAPHIC REFEREE.

No syntax errors found. CIF dictionary Interpreting this report

Datablock: 10a

```
Bond precision:  C-C = 0.0036 A          Wavelength=0.71073

Cell:           a=20.9331(9)    b=6.0827(3)         c=15.2530(11)
                alpha=90        beta=132.900(1)     gamma=90
Temperature:    100 K

                Calculated                Reported
Volume          1422.72(14)               1422.72(14)
Space group     C 2/c                     C2/c
Hall group      -C 2yc                    ?
Moiety formula  C10 H16 Br4 Se            C10 H16 Br4 Se
Sum formula     C10 H16 Br4 Se            C10 H16 Br4 Se
Mr              534.79                    534.83
Dx,g cm-3       2.497                     2.497
Z               4                         4
Mu (mm-1)       13.842                    13.843
F000            1000.0                    1000.0
F000’           996.10
h,k,lmax        29,8,21                   29,8,21
Nref            2094                      2090
Tmin,Tmax       0.074,0.575               0.158,0.601
Tmin’           0.044

Correction method= # Reported T Limits: Tmin=0.158 Tmax=0.601
AbsCorr = MULTI-SCAN

Data completeness= 0.998          Theta(max)= 30.060

R(reflections)= 0.0183( 1939)     wR2(reflections)= 0.0405( 2090)

S = 1.103                  Npar= 69
```

The following ALERTS were generated. Each ALERT has the format
test-name_ALERT_alert-type_alert-level.
Click on the hyperlinks for more details of the test.

Alert level C

```
ABSTY02_ALERT_1_C  An _exptl_absorpt_correction_type has been given without
            a literature citation. This should be contained in the
            _exptl_absorpt_process_details field.
            Absorption correction given as multi-scan
PLAT480_ALERT_4_C Long H...A H-Bond Reported H1       ..BR2      .       3.04 Ang.
PLAT480_ALERT_4_C Long H...A H-Bond Reported H2       ..BR1      .       3.11 Ang.
PLAT480_ALERT_4_C Long H...A H-Bond Reported H3B      ..BR1      .       2.98 Ang.
PLAT480_ALERT_4_C Long H...A H-Bond Reported H3A      ..BR2      .       3.05 Ang.
PLAT480_ALERT_4_C Long H...A H-Bond Reported H5A      ..SE1      .       3.05 Ang.
```

Alert level G

```
PLAT005_ALERT_5_G No Embedded Refinement Details Found  in the CIF     Please Do !
PLAT093_ALERT_1_G No s.u.'s on H-positions, Refinement Reported as      mixed Check
PLAT128_ALERT_4_G Alternate Setting for Input Space Group     C2/c       I2/a Note
PLAT434_ALERT_2_G Short Inter HL..HL Contact Br2      ..Br2              3.45 Ang.
                                                 1-x,-y,2-z  =      5_657 Check
PLAT764_ALERT_4_G Overcomplete CIF Bond List Detected (Rep/Expd) .       1.11 Ratio
PLAT793_ALERT_4_G Model has Chirality at C1          (Centro SPGR)          S Verify
PLAT793_ALERT_4_G Model has Chirality at C2          (Centro SPGR)          S Verify
PLAT899_ALERT_4_G SHELXL97   is Deprecated and Succeeded by SHELXL       2018 Note
```

```
   0 ALERT level A = Most likely a serious problem - resolve or explain
   0 ALERT level B = A potentially serious problem, consider carefully
   6 ALERT level C = Check. Ensure it is not caused by an omission or oversight
   8 ALERT level G = General information/check it is not something unexpected

   2 ALERT type 1 CIF construction/syntax error, inconsistent or missing data
   1 ALERT type 2 Indicator that the structure model may be wrong or deficient
   0 ALERT type 3 Indicator that the structure quality may be low
  10 ALERT type 4 Improvement, methodology, query or suggestion
   1 ALERT type 5 Informative message, check
```

It is advisable to attempt to resolve as many as possible of the alerts in all categories. Often the minor alerts point to easily fixed oversights, errors and omissions in your CIF or refinement strategy, so attention to these fine details can be worthwhile. In order to resolve some of the more serious problems it may be necessary to carry out additional measurements or structure refinements. However, the purpose of your study may justify the reported deviations and the more serious of these should normally be commented upon in the discussion or experimental section of a paper or in the "special_details" fields of the CIF. checkCIF was carefully designed to identify outliers and unusual parameters, but every test has its limitations and alerts that are not important in a particular case may appear. Conversely, the absence of alerts does not guarantee there are no aspects of the results needing attention. It is up to the individual to critically assess their own results and, if necessary, seek expert advice.

Publication of your CIF in IUCr journals

A basic structural check has been run on your CIF. These basic checks will be run on all CIFs submitted for publication in IUCr journals (*Acta Crystallographica*, *Journal of Applied Crystallography*, *Journal of Synchrotron Radiation*); however, if you intend to submit to *Acta Crystallographica Section C* or *E* or *IUCrData*, you should make sure that full publication checks are run on the final version of your CIF prior to submission.

Publication of your CIF in other journals

Please refer to the *Notes for Authors* of the relevant journal for any special instructions relating to CIF submission.

PLATON version of 07/08/2019; check.def file version of 30/07/2019

Datablock 10a - ellipsoid plot

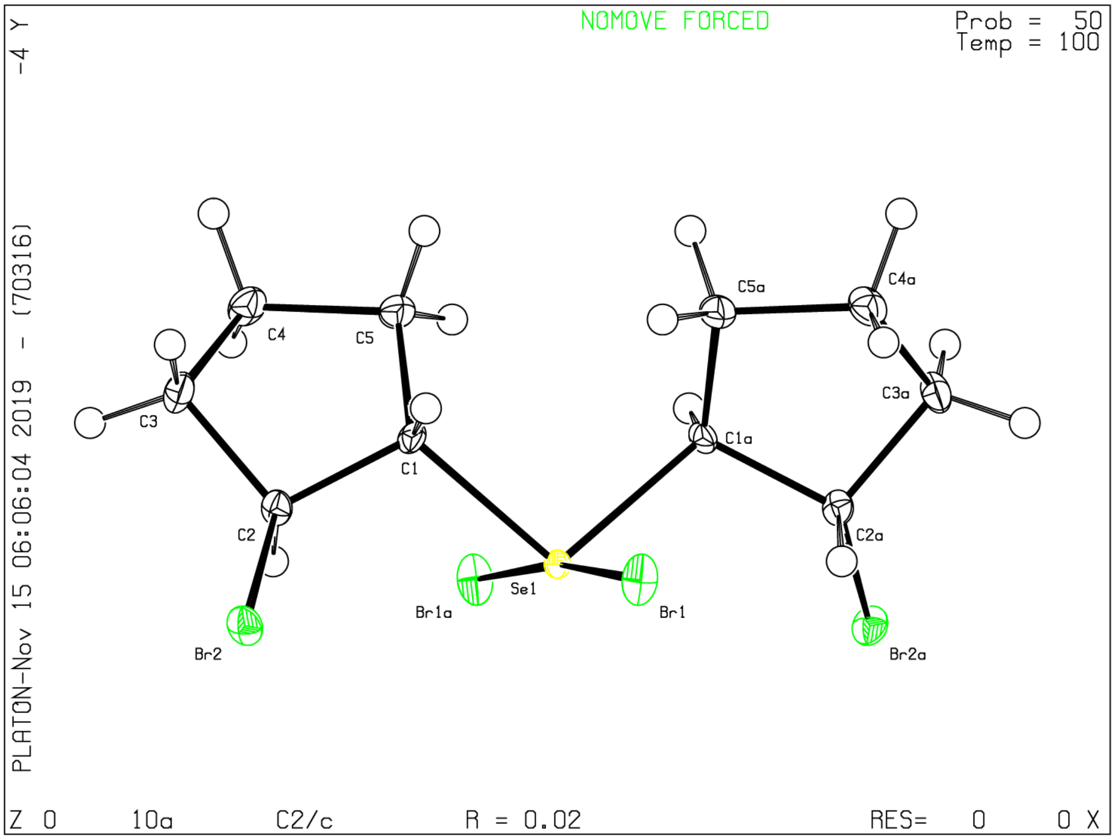

Supplement: Supplementary file 1 [file molecules-25-00194-s001.zip › molecules-663620/checkcif_10a.pdf]
