# Supplementary material for: Remarkable Alkene-to-Alkene and Alkene-to-Alkyne Transfer Reactions of Selenium Dibromide and PhSeBr. Stereoselective Addition of Selenium Dihalides to Cycloalkenes
Source: Molecules. 2020 Jan 3;25(1):194. doi: 10.3390/molecules25010194 (PMC6983007; doi:10.3390/molecules25010194)
Supplement: Supplementary file 1 [file molecules-25-00194-s001.zip › molecules-663620/molecules-663620.pdf]

Remarkable alkene-to-alkene and alkene-to-alkyne transfer reactions of selenium dibromide and PhSeBr. Stereoselective addition of selenium dihalides to cycloalkenes.

Vladimir A. Potapov,* Maxim V. Musalov, Evgeny O. Kurkutov, Vladimir A. Yakimov, Alfiya G. Khabibulina, Maria V. Musalova, Svetlana V. Amosova, Tatyana N. Borodina, Alexander I. Albanov

A. E. Favorsky Irkutsk Institute of Chemistry, Siberian Division of The Russian Academy of Sciences, 1 Favorsky Str., Irkutsk 664033, Russian Federation; v.a.potapov@mail.ru

Table of Contents

Experimental (General)	2
Synthesis of compounds **12-16a**,**b** and monitoring data	3-7
Examples of NMR spectra of the obtained compounds	8-25
X-ray crystallographic study of compounds **9a** and **10a**	26-27

Experimental

General

X-ray diffraction experiments were carried out on a Bruker D8 Venture Photon 100 CMOS diffractometer with Mo-K_α radiation (λ = 0.71073 Å). ^{1}H (400.1 MHz) and ^{13}C (100.6 MHz) NMR spectra were recorded on a Bruker DPX-400 spectrometer in 5-10% solution in $CDCl_3$. ^{1}H and ^{13}C chemical shifts (δ) are reported in parts per million (ppm), relative to the residual solvent peak of $CDCl_3$ (δ = 7.27 and 77.00 ppm in ^{1}H and ^{13}C-NMR, respectively). All coupling constants (*J* values) were reported in Hertz (Hz). The following abbreviations were used to explain multiplicities: s = singlet, d = doublet, t = triplet, q = quartet, m= multiplet. Mass spectra were recorded on a Shimadzu GCMS-QP5050A with electron impact (EI) ionization at 70 eV. Elemental analysis was performed on a Thermo Scientific Flash 2000 Elemental Analyzer.

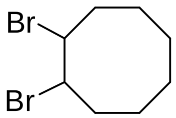

11

1,2-Dibromocyclooctane (11) was isolated in 40% yield by column chromatography (silica gel, hexane → hexane/chloroform 9 : 1) from the reaction of selenium dibromide with cyclooctene in acetonitrile. ^{1}H NMR (400.1 MHz, $CDCl_3$): 1.42-1.45 (m, 2H, CH_2), 1.53-1.65 (m, 4H, CH_2), 1.79-1.81 (m, 2H, CH_2), 2.02-2.07 (m, 2H, CH_2), 2.33-2.40 (m, 2H, CH_2), 4.53-4.54 (m, 2H, CHBr). ^{13}C NMR (100.6 MHz, $CDCl_3$): δ 25.19 (CH_2), 25.73 (CH_2), 33.02 (CH_2), 61.31 (CHBr). Anal. Calcd for $C_8H_{14}Br_2$: C, 35.59; H, 5.23; Br, 59.19. Found, %: C, 35.87; H, 5.41; Br, 58.93.

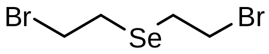

12

Bis(2-bromoethyl) selenide (12). Dry ethylene was bubbled to a flask containing CCl_4 (20 mL) with stirring for 20 min at room temperature. A solution of $SeBr_2$ [(5.75 mmol, prepared from elemental selenium (0.454 g) and bromine (0.920 g) in CCl_4 (20 mL)] was added dropwise to the flask for 40 min with stirring. The ethylene bubbling (~30 mL/min) was continued during the $SeBr_2$ addition and 20 min after the addition. The mixture was stirred additionally for 2 h at room temperature and filtered. The solvent was removed in vacuum giving selenide **12** (1.662 g, 98% yield) as a yellowish white powder, mp 44-45 °C. ^{1}H-NMR (400 MHz, $CDCl_3$): δ 3.63–3.54 (m, 4H), 3.09–3.01 (m, 4H). ^{13}C-NMR (100 MHz, $CDCl_3$): δ 30.68, 25.56.

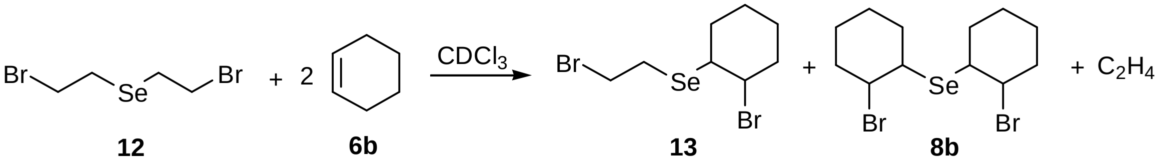

1-Bromo-2-[(2-bromoethyl)selanyl]cyclohexane (13). A solution of cyclohexene (0.021 g, 0.25 mmol) in $CDCl_3$ (0.3 mL) was added to a solution of selenide **12** (0.037 g, 0.125 ммоль) in $CDCl_3$ (0.3 mL). The mixture was monitored by NMR spectroscopy.

The molar ratios of the compounds **12** : **13** : **8b** were 50 : 46 : 4 (4 days), 34 : 51 : 15 (10 days), 20 : 53 : 27 (18 days), 1 : 49 : 50 (28 days). The compound **13** was characterized in the mixture with compound **8b**. ^{1}H-NMR (400 MHz, $CDCl_3$): δ 1.45-1.68 (m, 2H, CH_2), 1.70-1.85 (m, 2H, CH_2), 1.88-2.07 (m, 2H, CH_2), 2.29-2.48 (m, 2H, CH_2), 3.07-3.12 (m, 2H, CH_2Se), 3.32-3.39 (m, 1H, CHSe), 3.58-3.65 (m, 2H, CH_2Br), 4.46-4.54 (m, 1H, CHBr). ^{13}C-NMR (100 MHz, $CDCl_3$): δ 22.57 (CH_2), 23.65 (CH_2), 26.15 (CH_2Se), 30.24 (CH_2), 30.86 (CH_2Br), 33.06 (CH_2), 46.49 (CHSe), 57.49 (CHBr).

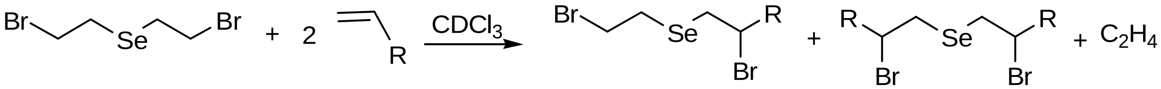

12 **1a,c** **14a,c** **5a,c**

R = C_4H_9 (**a**), C_6H_{13} (**c**)

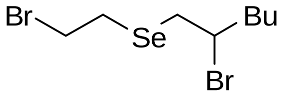

14a

2-Bromo-1-[(2-bromoethyl)selanyl]hexane (14a). A solution of 1-hexene (0.02 g, 0.24 mmol) in $CDCl_3$ (0.3 mL) was added to a solution of selenide **12** (0.035 g, 0.12 ммоль) in $CDCl_3$ (0.3 mL). The mixture was monitored by NMR spectroscopy. The molar ratios of the compounds **12** : **14a** : **5a** were 18 : 50 : 32 (4 days), 7 : 49 : 44 (9 days), 0 : 46 : 54 (14 days). The compound **14a** was characterized in the mixture with compound **5a**. ^{1}H-NMR (400 MHz, $CDCl_3$): δ 0.93 (t, 3H, CH_3), 1.28-1.47 (m, 3H, CH_2), 1.51-1.61 (m, 2H, CH_2), 1.73-1.83 (m, 1H, CH_2), 1.98-2.08 (m, 1H, CH_2), 3.03-3.12 (m, 2H, CH_2Se), 3.20-3.26 (m, 1H, CH_2Se), 3.51-3.61 (m, 2H, CH_2Br), 4.12-4.20 (m, 1H, CHBr). ^{13}C-NMR (100 MHz, $CDCl_3$): δ 13.87 (CH_3), 21.97(CH_2), 26.35 (CH_2Se), 29.48 (CH_2), 30.73 (CH_2Br), 33.31 (CH_2Se), 37.47(CH_2), 55.53 (CHBr).

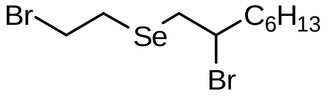

14c

2-Bromo-1-[(2-bromoethyl)selanyl]octane (14c). A solution of 1-octene (0.027 g, 0.12 mmol) in $CDCl_3$ (0.3 mL) was added to a solution of selenide **12** (0.035 g, 0.12 ммоль) in $CDCl_3$ (0.3 mL). The mixture was monitored by NMR spectroscopy. The

molar ratios of the compounds **12** : **14c** : **5c** were 19 : 51 : 31 (6 days), 6 : 50 : 44 (15 days), 1 : 50 : 49 (20 days).

The compound **14c** was characterized in the mixture with compound **5c**. ^{1}H-NMR (400 MHz, $CDCl_3$): δ ^{13}C-NMR (100 MHz, $CDCl_3$): δ 0.93(t, 3H, CH_3), 1.25-1.50 (m, 7H, CH_2), 1.53-1.61 (m, 1H, CH_2), 1.75-1.84 (m, 1H, CH_2), 2.02-2.08 (m, 1H, CH_2), 3.04-3.16 (m, 2H, CH_2Se), 3.20-3.27 (m, 1H, CH_2Se), 3.57-3.61 (m, 2H, CH_2Br), 4.15-4.21 (m, 1H, CHBr). Спектр ЯМР ^{13}C, δ, м.д.: 14.05 (CH_3), 22.54(CH_2), 26.32 (CH_2Se), 27.32 (CH_2), 28.53 (CH_2), 30.72 (CH_2Br), 31.60 (CH_2), 33.28 (CH_2Se), 37.71 (CH_2), 55.58 (CHBr).

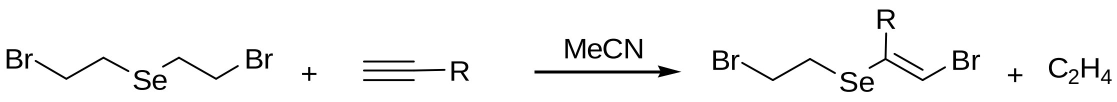

12 **15a,b**

R = C_4H_9 (**a**), C_5H_{11} (**b**)

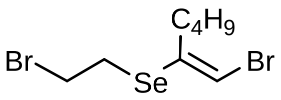

15a

(1*E*)-1-bromo-2-[(2-bromoethyl)selanyl]hex-1-ene (15a). A solution of 1-hexyne (0.082 g, 1 mmol) in MeCN (1 mL) was added to a solution of selenide **12** (0.1 g, 0.34 mmol) in MeCN (1.5 mL) and the mixture was stirred in a 200 mL round-bottomed closed flask for 30 h at room temperature. The solvent was removed by a rotary evaporator. The residue contained compounds **15a** (0.107 g, 90% yield) and unconverted selenide **12** in a 9 : 1 molar ratio (the NMR data). The compound **15a** was characterized in the mixture with selenide **12**. ^{1}H-NMR (400 MHz, $CDCl_3$): δ 6.34 (s, 1H), 3.47–3.43 (m, 2H), 3.02–3.98 (m, 2H), 2.38–2.34 (m, 2H), 1.58–1.54 (m, 2H), 1.47–1.37 (m, 2H), 1.31–1.22 (m, 2H), 0.86–0.82 (m, 3H). ^{13}C-NMR (100 MHz, $CDCl_3$): δ 133.14, 105.81, 34.72, 30.02, 29.37, 26.68, 21.72, 13.56. MS (EI): *m*/*z* (%) = 350 (20) [$M^{+\cdot}$], 306 (15), 269 (22), 199 (12), 161(15), 81 (100).

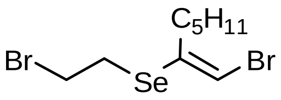

15b

(1*E*)-1-bromo-2-[(2-bromoethyl)selanyl]hept-1-ene (15b) was obtained in 92% yield from 1-heptyne and selenide **12** in acetonitrile under similar conditions as compound **15a.** The compound **15b** was characterized in the mixture with selenide **12**. ^{1}H-NMR (400 MHz, $CDCl_3$): δ 6.45 (s, 1H), 3.57–3.53 (m, 2H), 3.12–3.08 (m, 2H), 2.48–2.44 (m, 2H), 1.58–1.54 (m, 2H), 1.37–1.28 (m, 4H), 0.94–0.90 (m, 3H). ^{13}C-NMR (100 MHz, $CDCl_3$): δ 133.48, 106.27, 35.28, 31.06, 30.28, 27.25, 26.96, 22.40, 13.94. MS (EI): *m*/*z* (%) = 364 (20) [$M^{+\cdot}$], 306 (16), 227 (15), 199 (9), 95 (100).

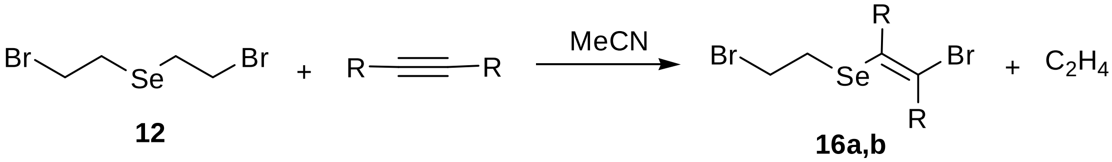

R = Et (**a**), Pr (**b**)

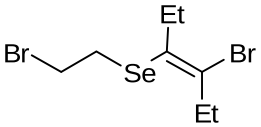

16a

(3*E*)-3-Bromo-4-[(2-bromoxyethyl)selanyl]hex-3-ene (16a) was obtained in 93% yield from 3-hexyne and selenide **12** in acetonitrile under the same conditions as compound **16b.** ^{1}H-NMR (400 MHz, $CDCl_3$): δ ^{1}H NMR (400 MHz, $CDCl_3$): δ 3.55–3.50 (m, 2H), 3.10–3.05 (m, 2H), 2.88–2.83 (m, 2H), 2.57–2.51 (m, 2H), 1.13–1.09 (m, 6H). ^{13}C NMR (100 MHz, $CDCl_3$): δ 130.73, 127.19, 35.26, 32.34, 30.70, 27.12, 13.45, 12.43.

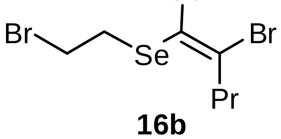

16b

(4*E*)-4-Bromo-5-[(2-bromoxyethyl)selanyl]oct-4-ene (16b). A solution of 4-octyne (0.11 g, 1 mmol) in MeCN (1 mL) was added to a solution of selenide **12** (0.1g, 0.34 mmol) in MeCN (1.5 mL) and the mixture was stirred in a 200 mL round-bottomed closed flask overnight (14 h) at room temperature. The solvent was removed by a rotary evaporator. The residue contained compounds **16b** (0.117 g, 91% yield) and **19b** in a 20 : 1 molar ratio (the NMR data).

The ^{1}H-NMR monitoring of the 16b and 19b formation from selenide 12 (CD_3CN).

The reaction of selenide **12** with 4-octyne (a 1 : 2 molar ratio) was monitored by NMR spectroscopy at room temperature using CD_3CN solution in a closed NMR ampoule. The decrease of the content of starting selenide **12** with proportional increasing the contents of compound **16b** and bis[(*E*)-2-bromo-1-propyl-1-pentenyl] selenide (**19b**) was observed. The formation of symmetrical selenide **19b** occurred by the reaction of compound **16b** with 4-octyne. After 145 h, the molar ratio of the compounds **12** : **16b** : **19b** was 34 : 51 : 15 (**Figure S1**).

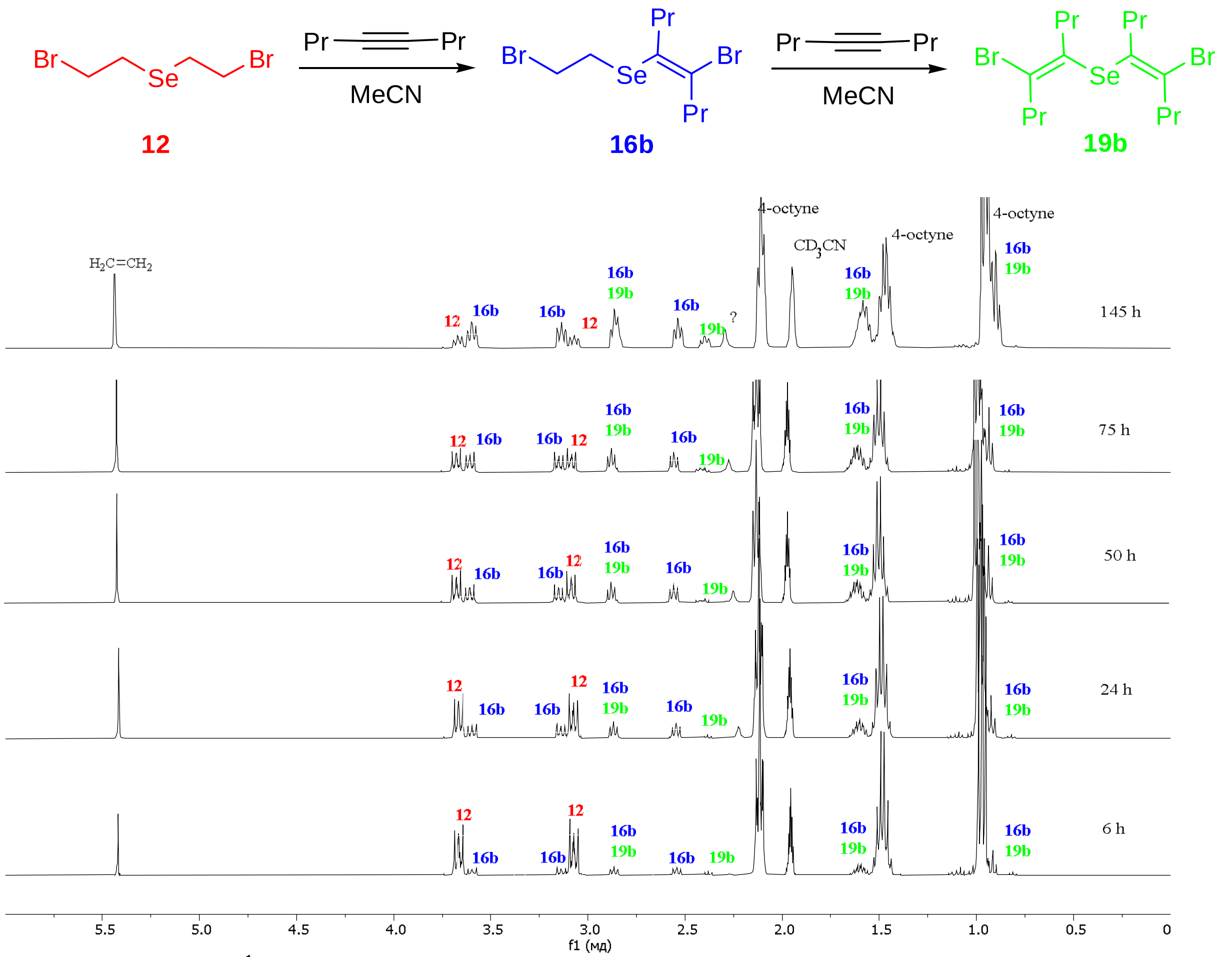

Figure S1. The ^{1}H-NMR monitoring of the **16b** and **19b** formation from selenide **12** (CD_3CN solution in a closed NMR ampoule).

Examples of ^{1}H- and ^{13}C-NMR spectra of the obtained compounds

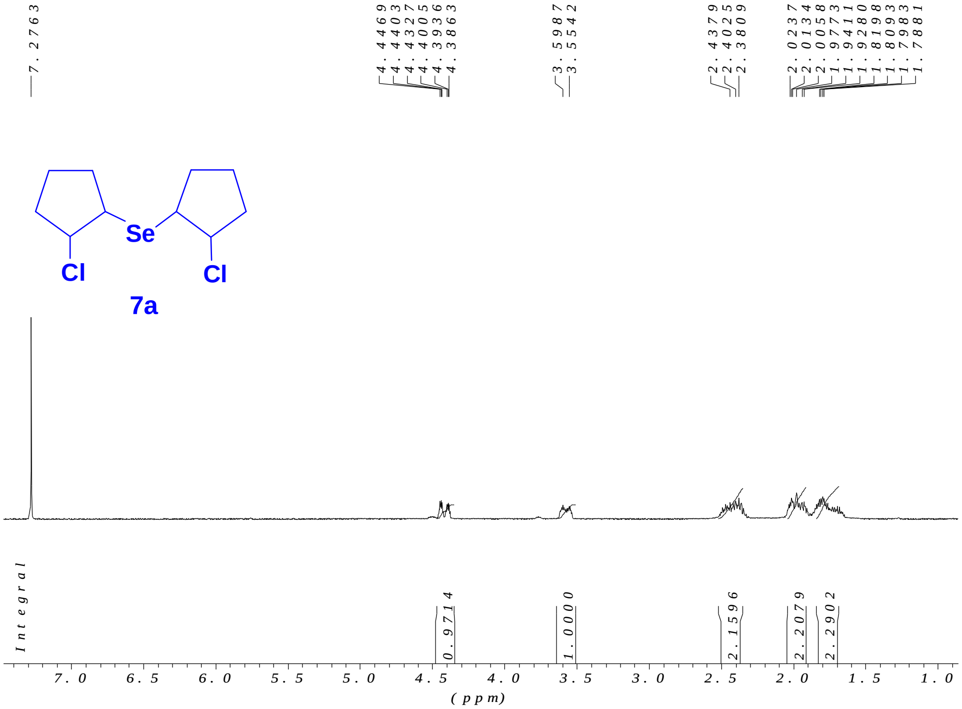

^{1}H-NMR spectrum of selenide 7a

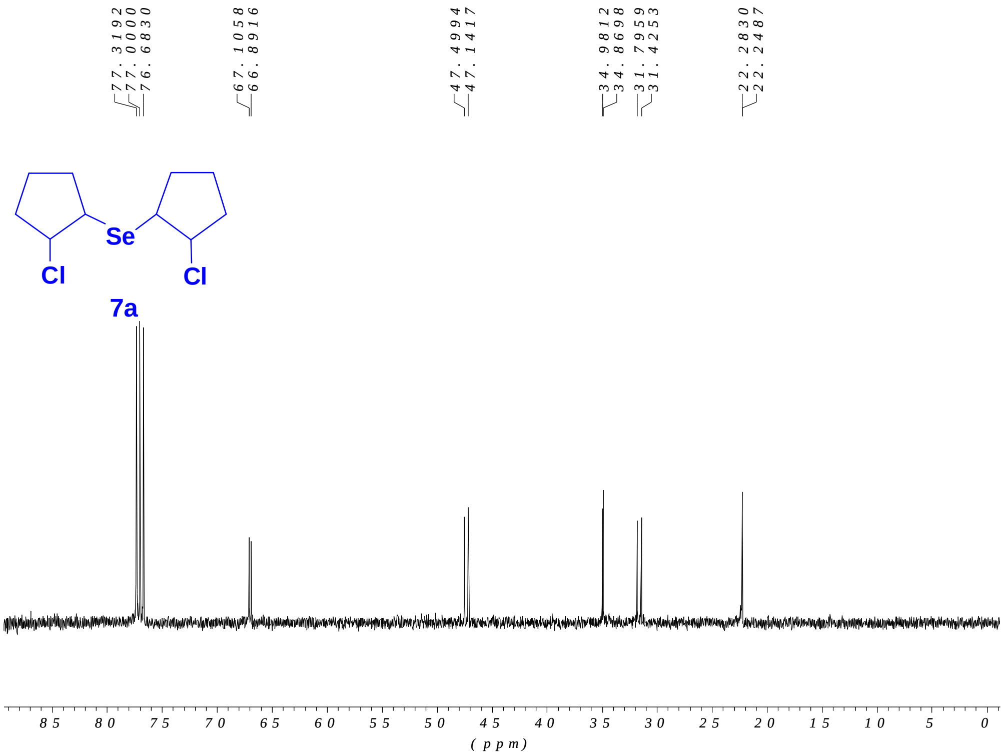

^{13}C-NMR spectrum of selenide 7a

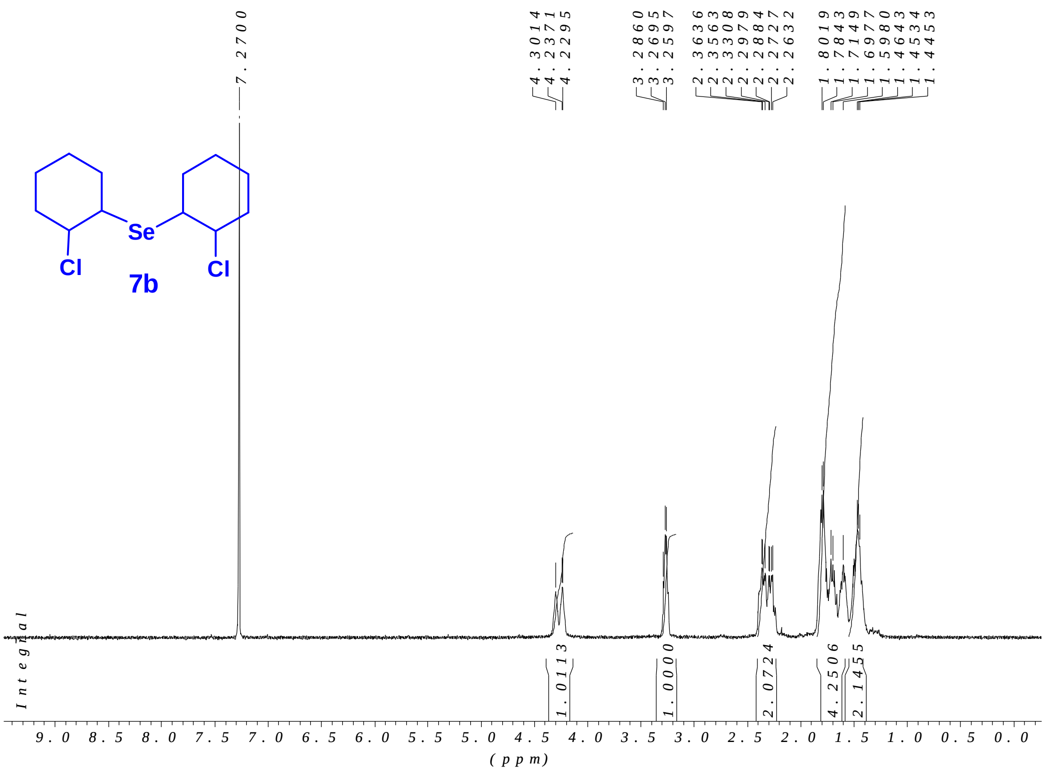

^{1}H-NMR spectrum of selenide 7b

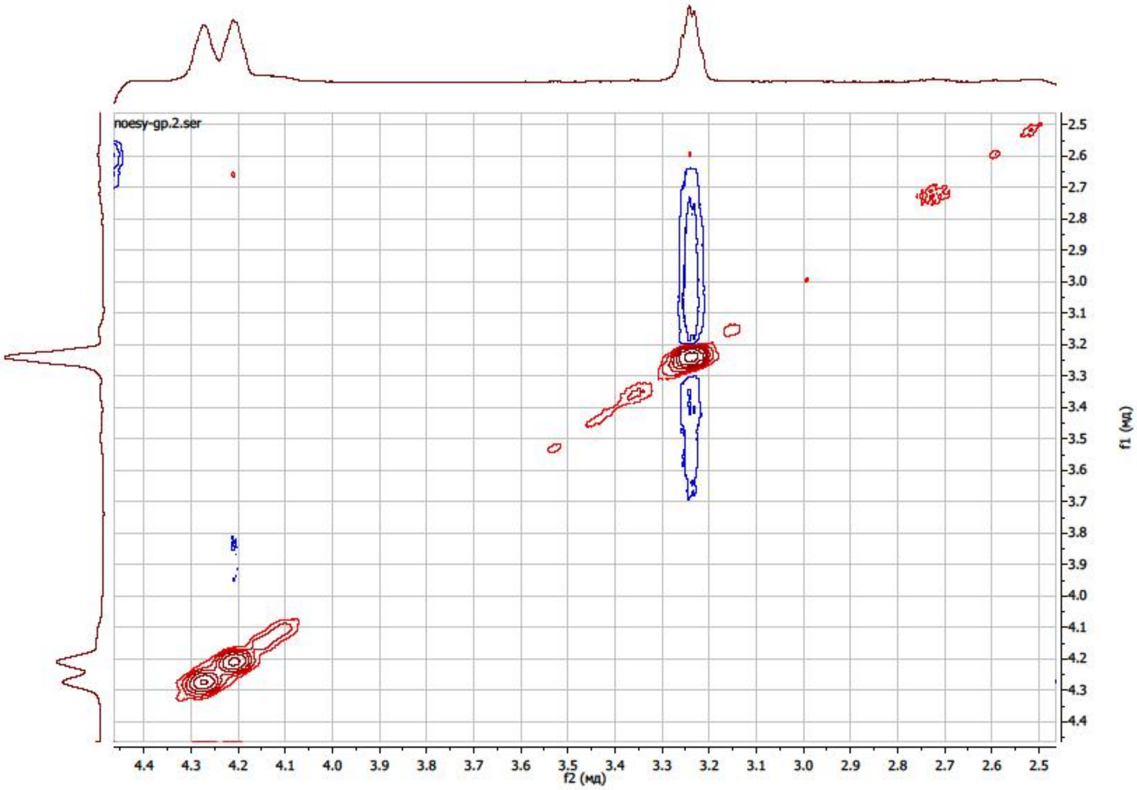

NMR NOESY spectrum of 7b (trans-disposition of the protons in the group SeCH-CHCl)

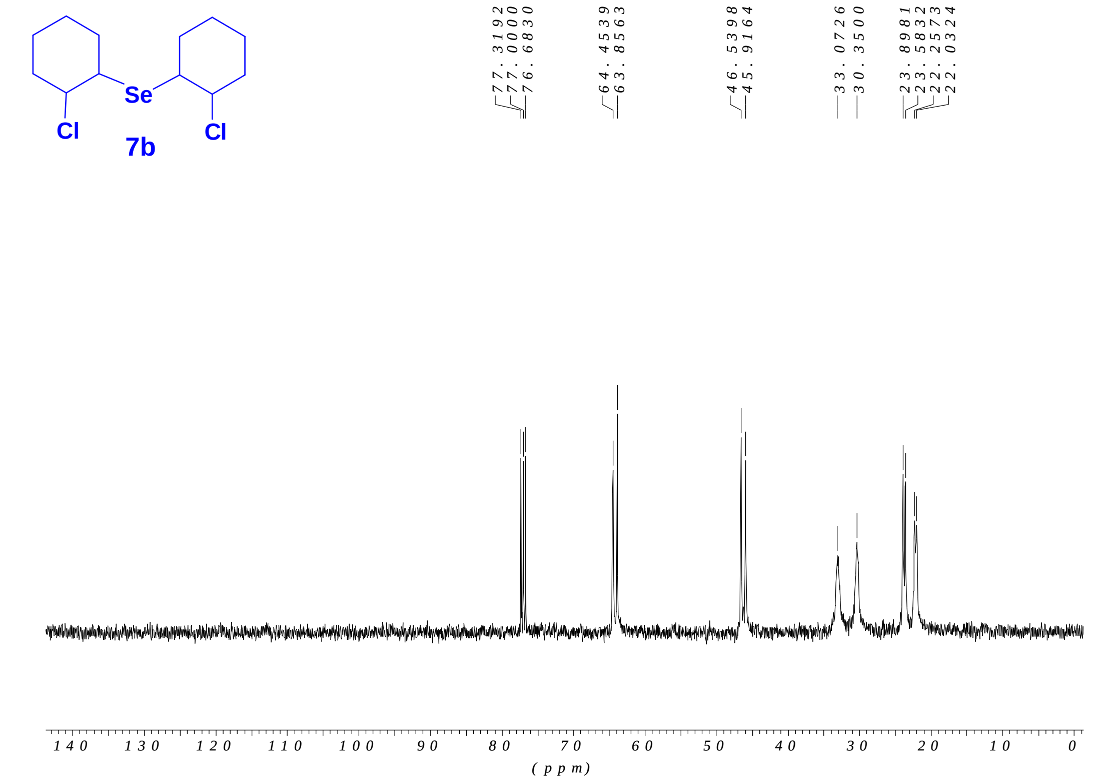

^{13}C-NMR spectrum of selenide 7b

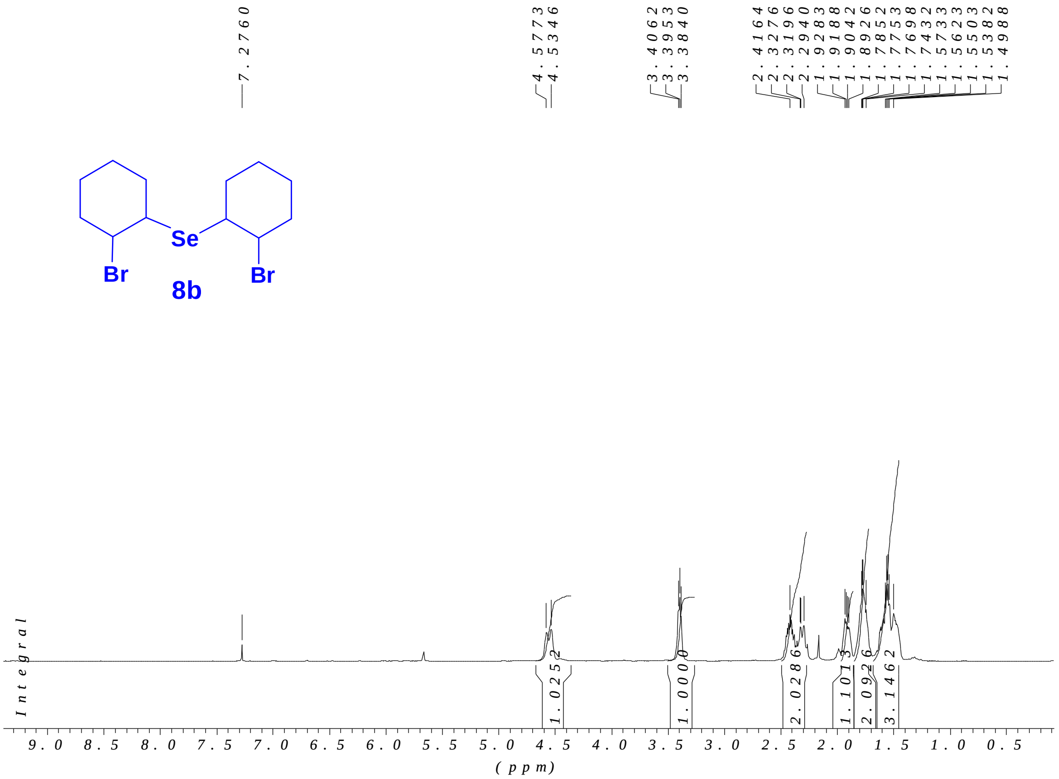

^{1}H-NMR spectrum of selenide 8b

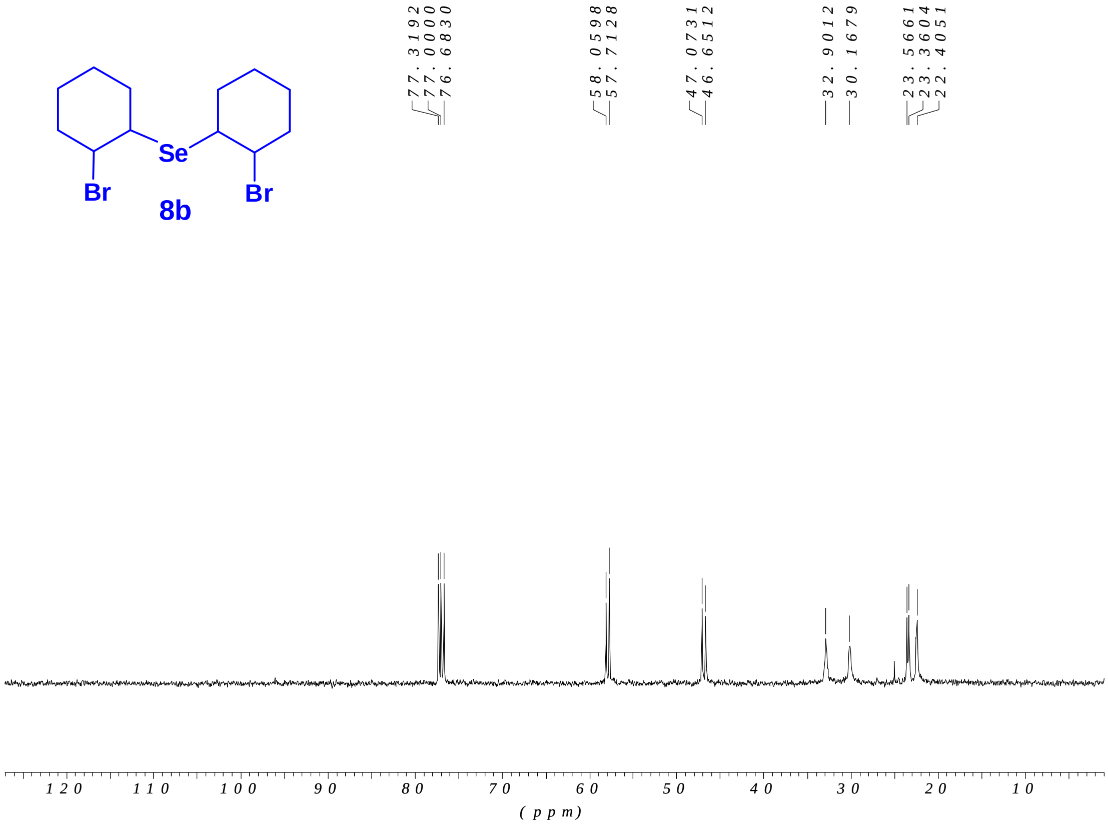

^{13}C-NMR spectrum of selenide 8b

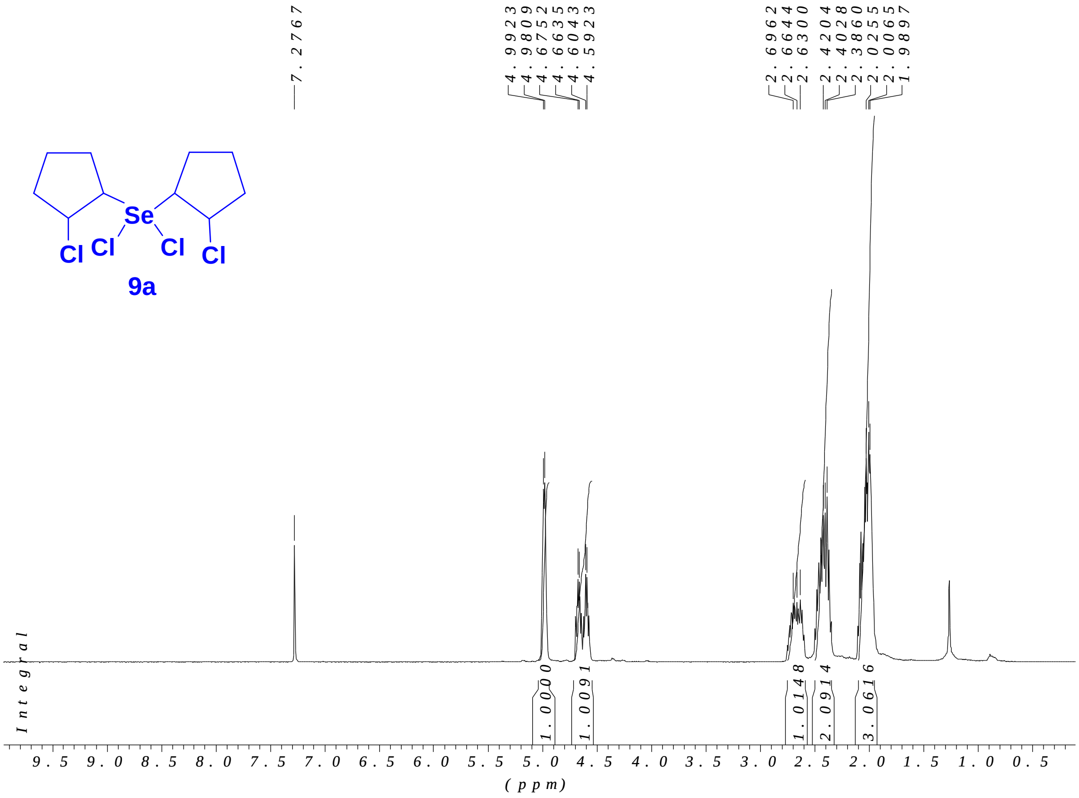

^{1}H-NMR spectrum of selane 9a

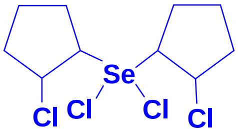

9a

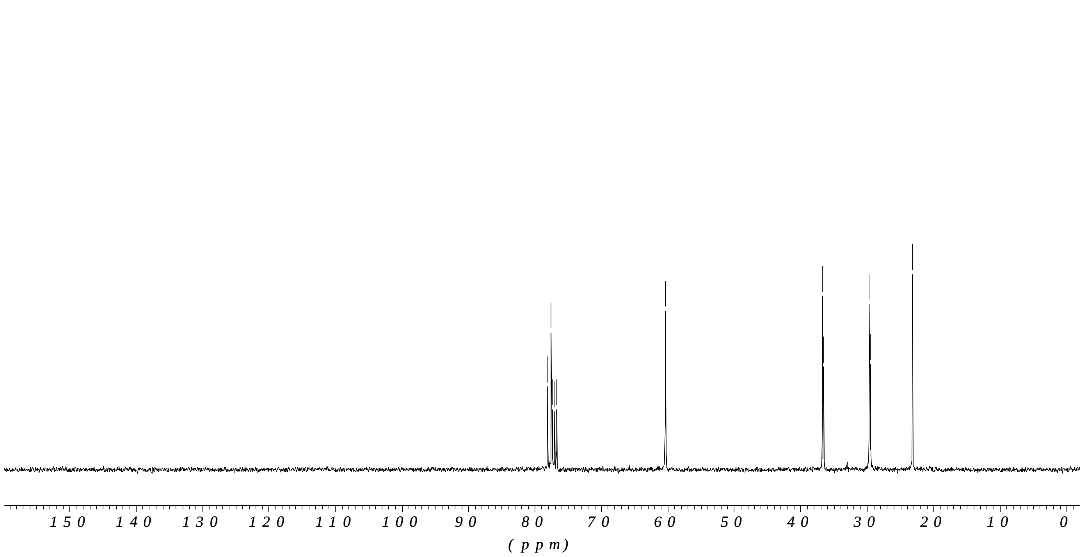

^{13}C-NMR spectrum of selane 9a

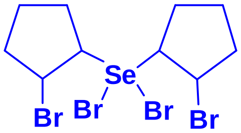

10a

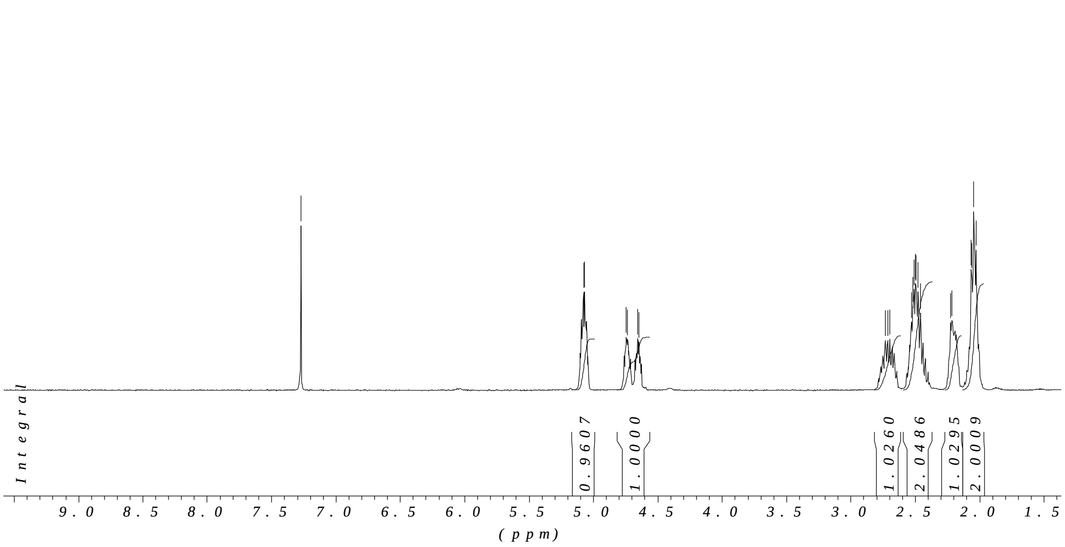

^{1}H-NMR spectrum of selane 10a

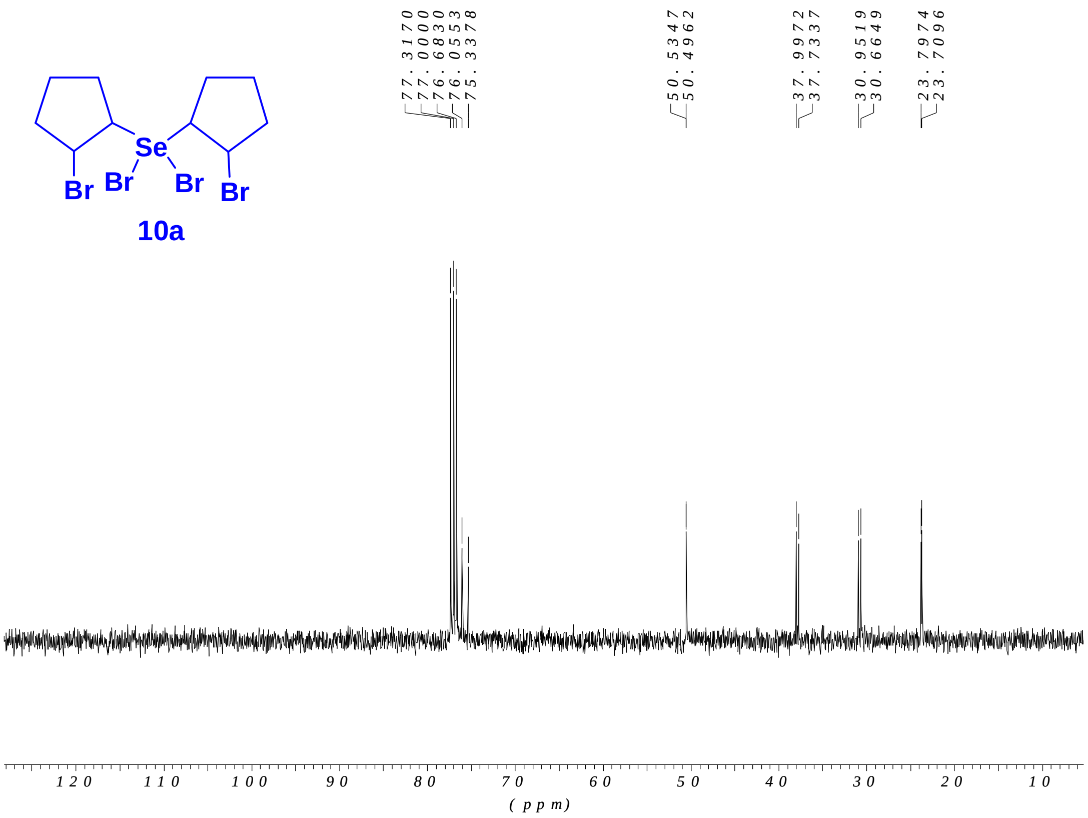

^{13}C-NMR spectrum of selane 10a

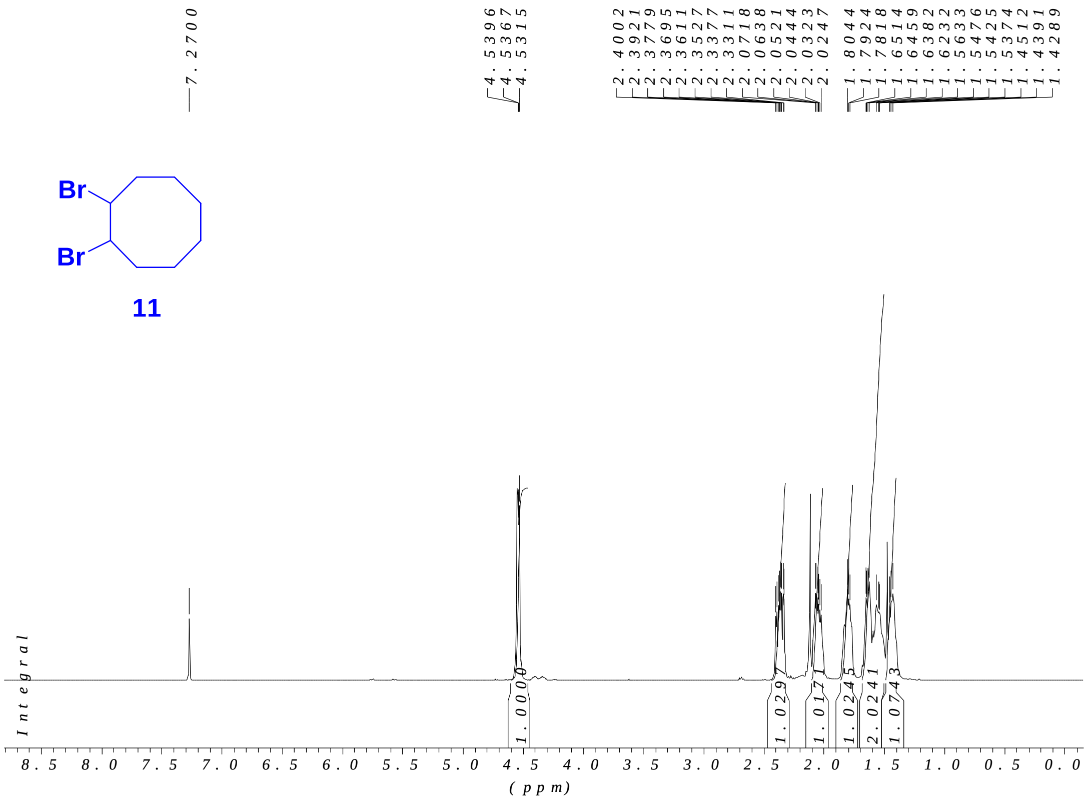

^{1}H-NMR spectrum of compound 11

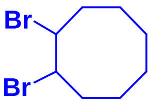

11

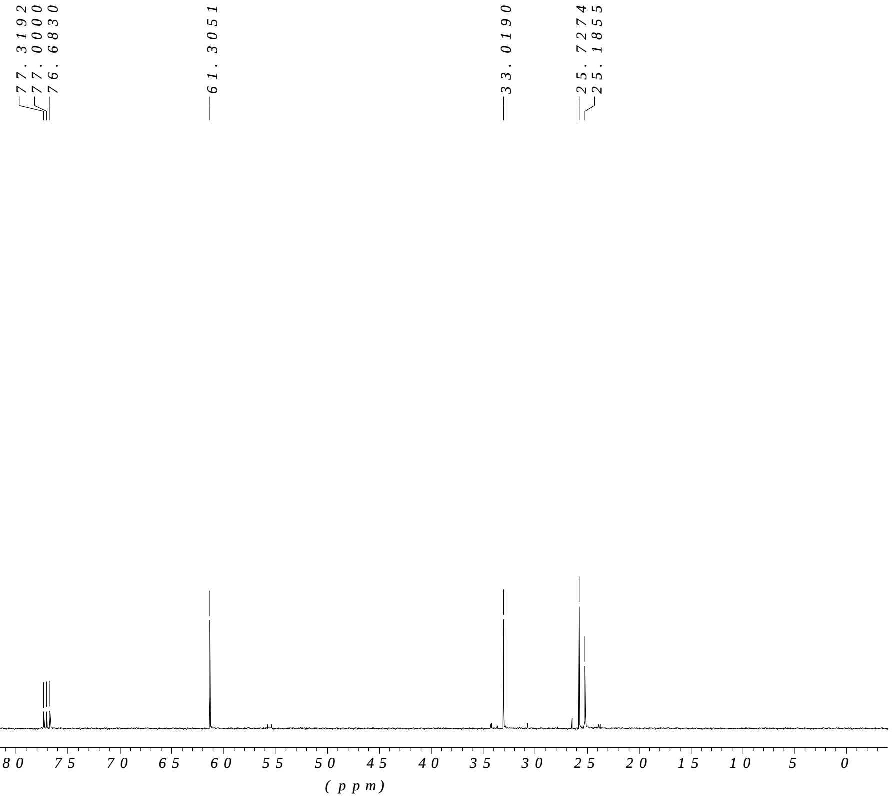

^{13}C-NMR spectrum of compound 11

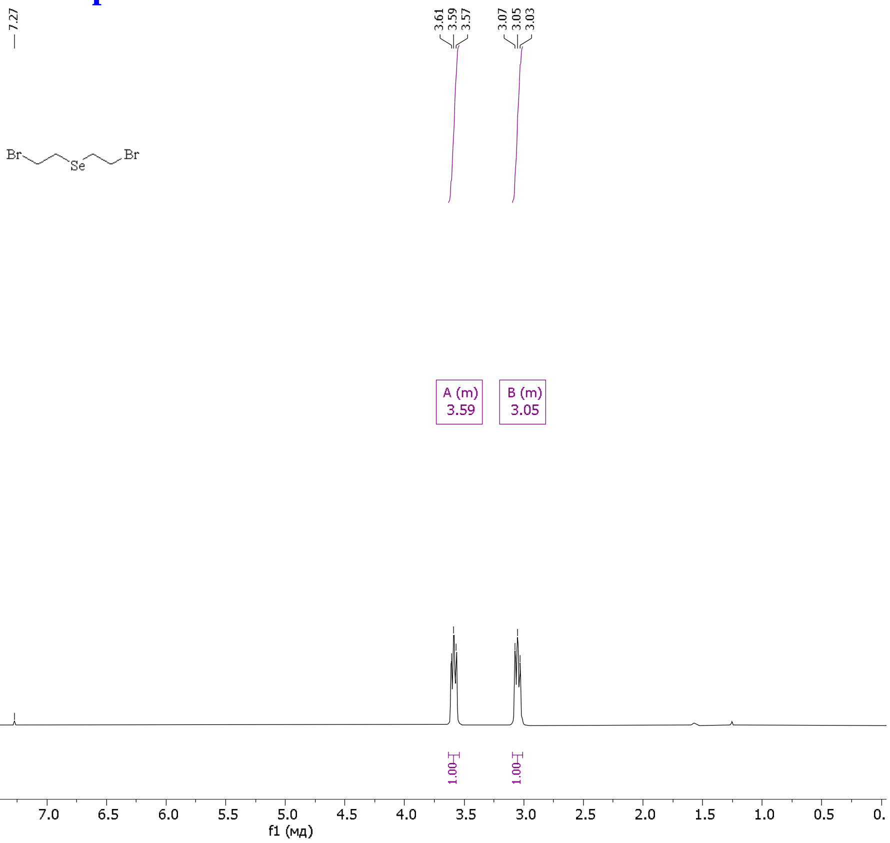

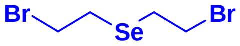

12

^{1}H-NMR spectrum of compound 12

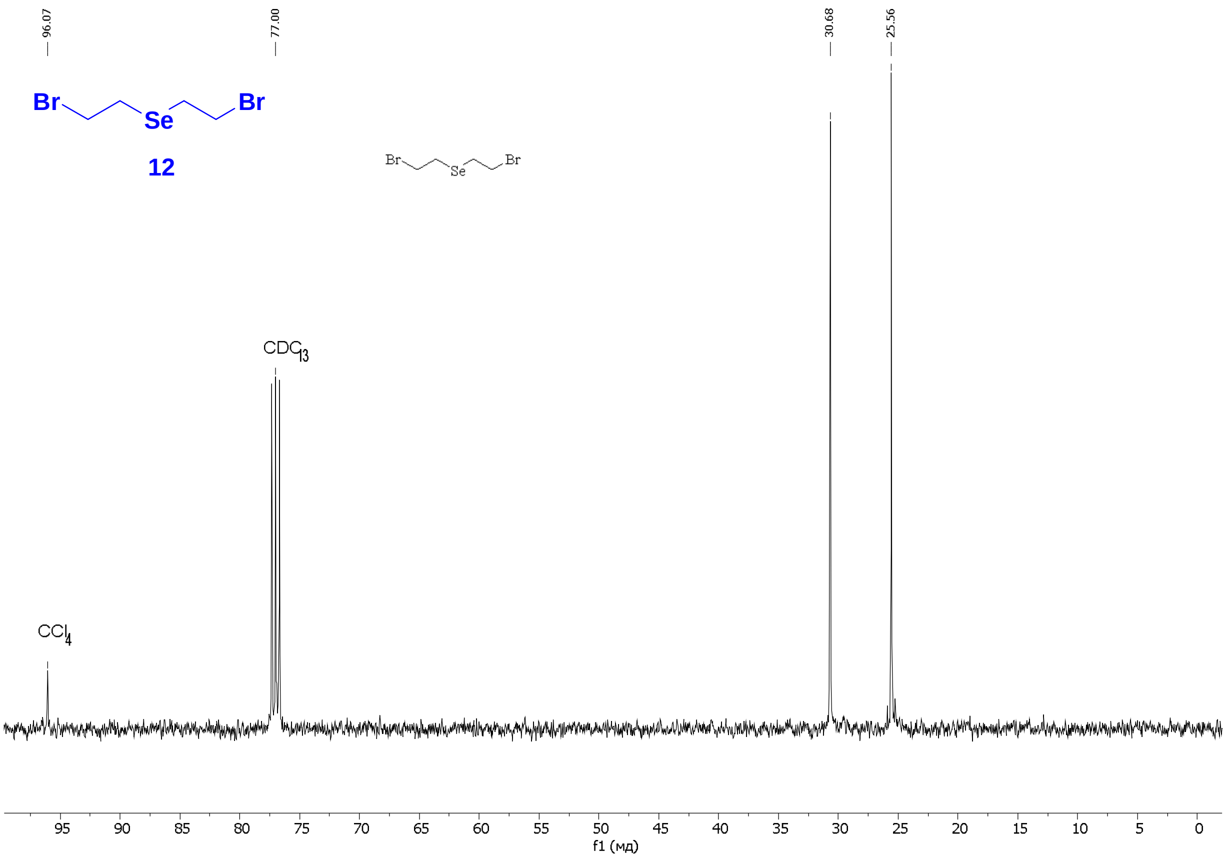

^{13}C-NMR spectrum of compound 12

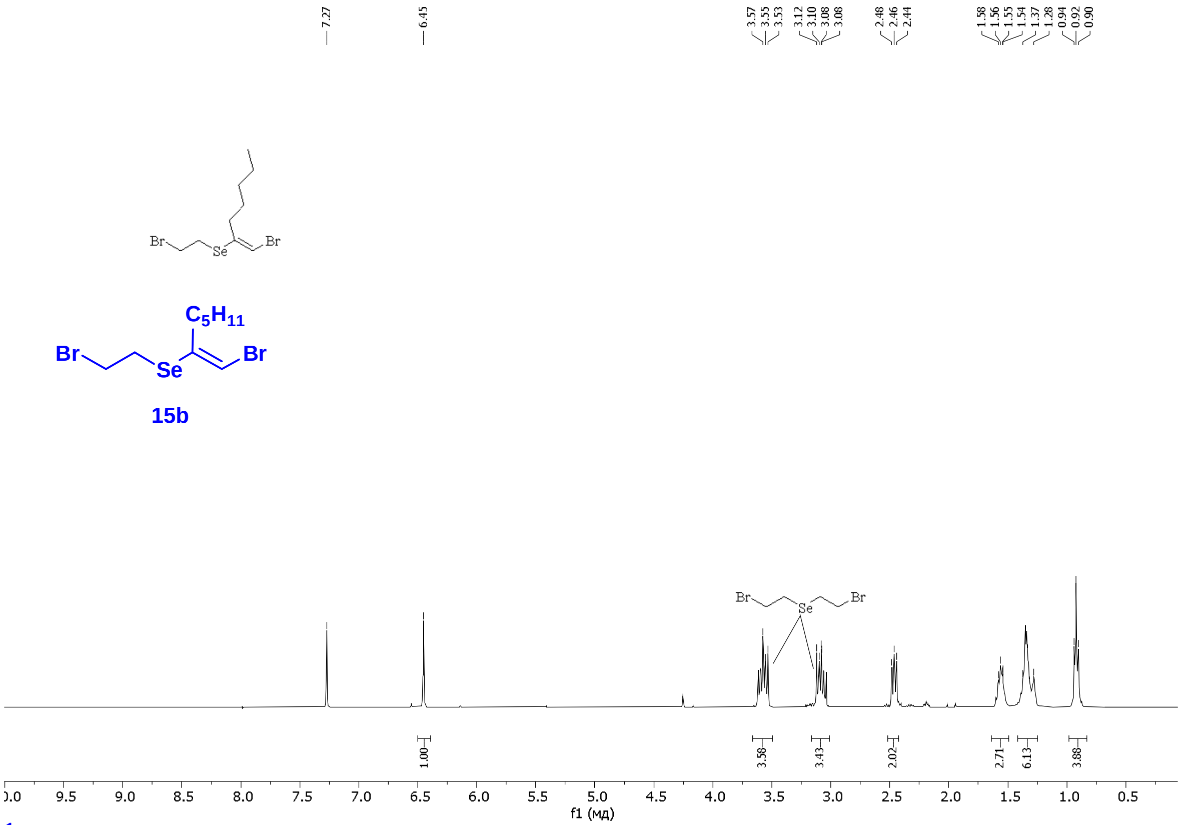

^{1}H-NMR spectrum of compound 15b (the admixture is compound 12)

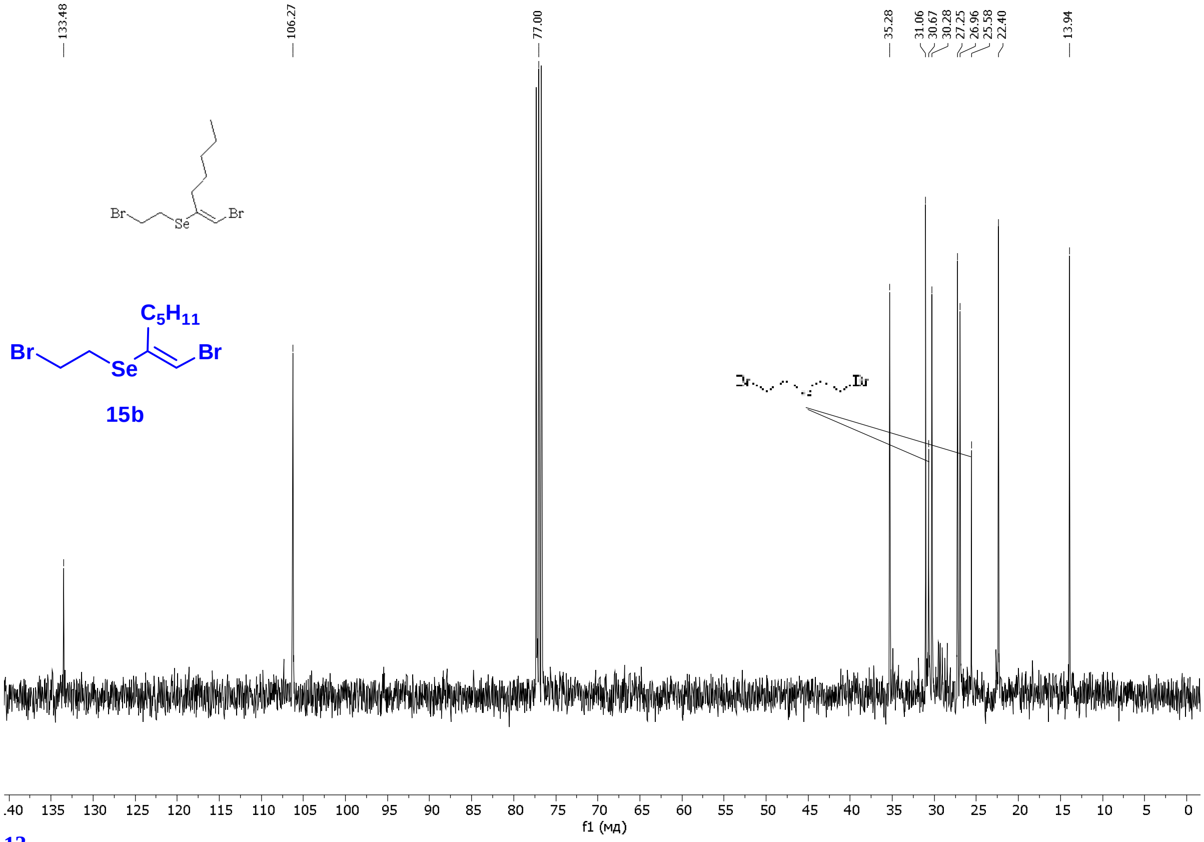

^{13}C-NMR spectrum of compound 15b (the admixture is compound 12)

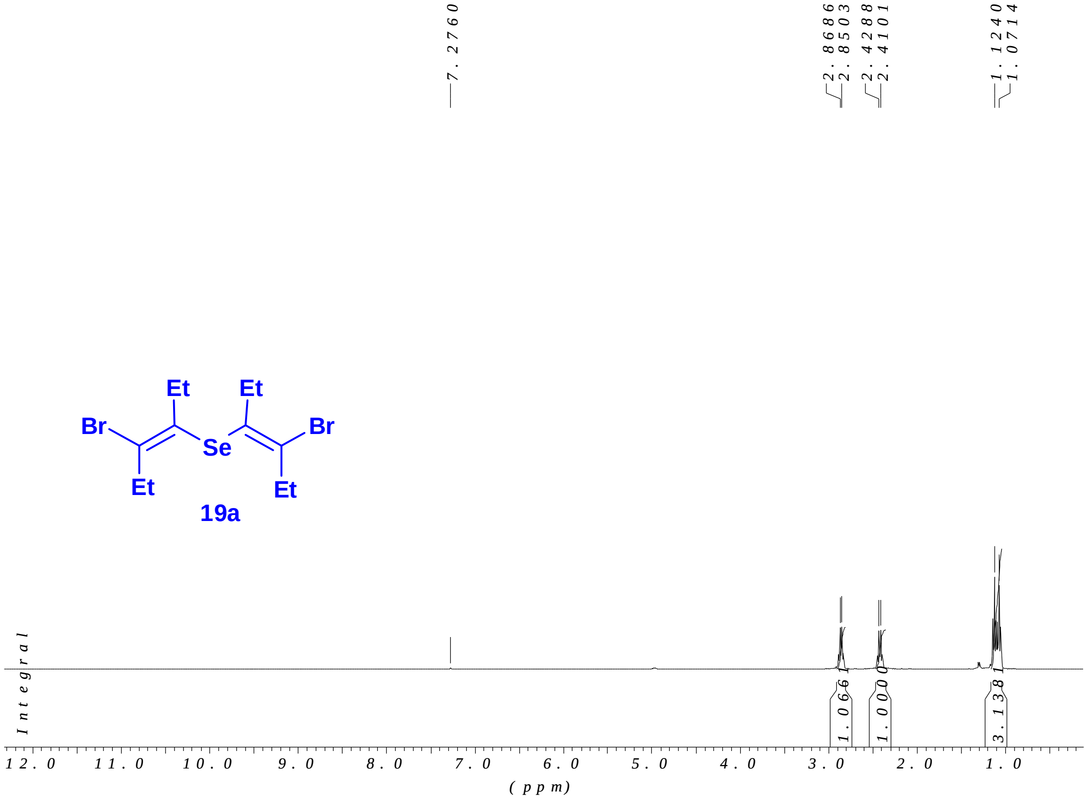

^{1}H-NMR spectrum of compound 19a

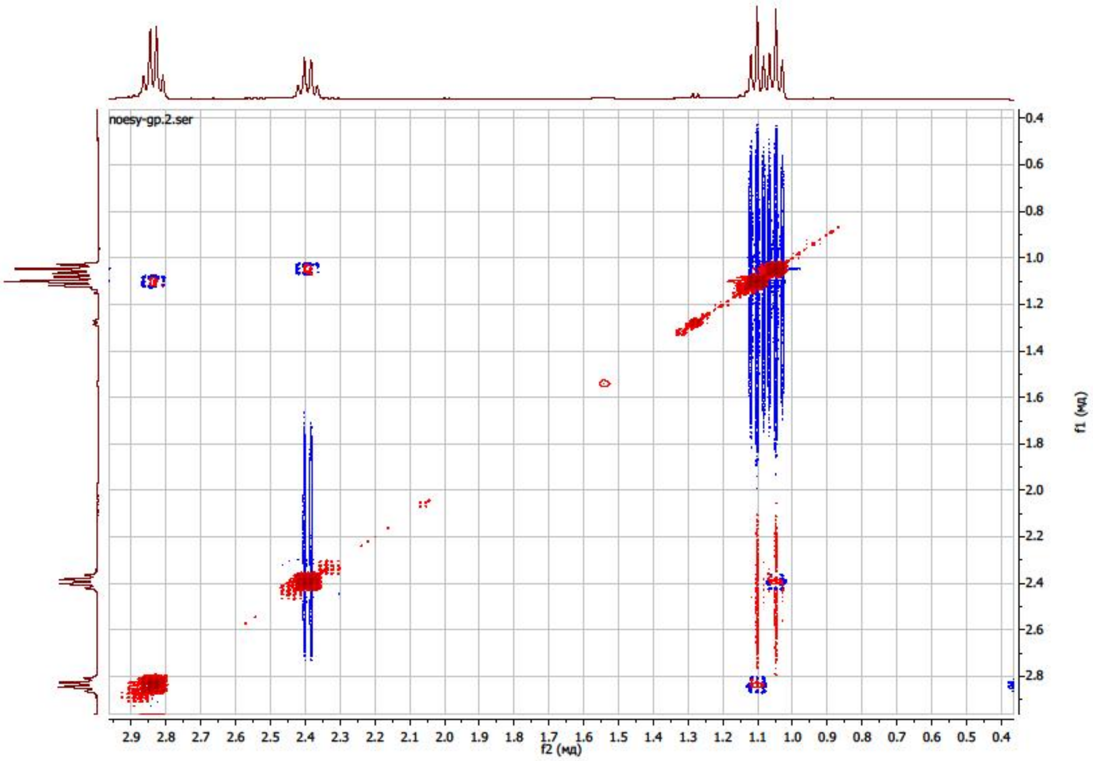

NMR NOESY spectrum of 19a (trans-disposition of two ethyl groups)

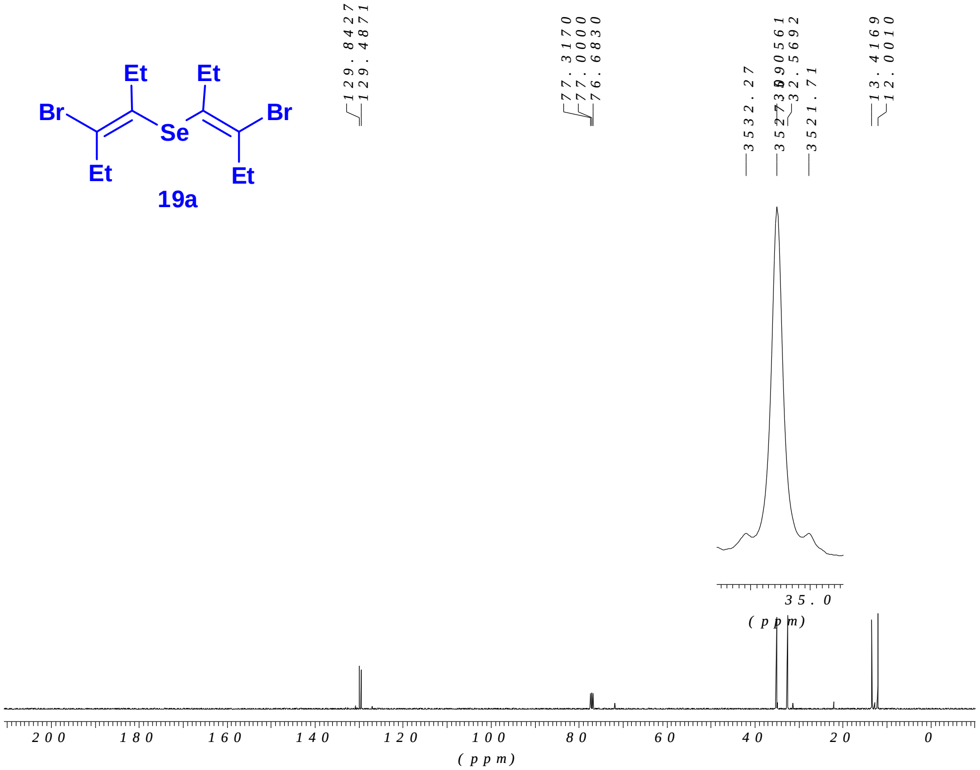

^{13}C-NMR spectrum of compound 19a

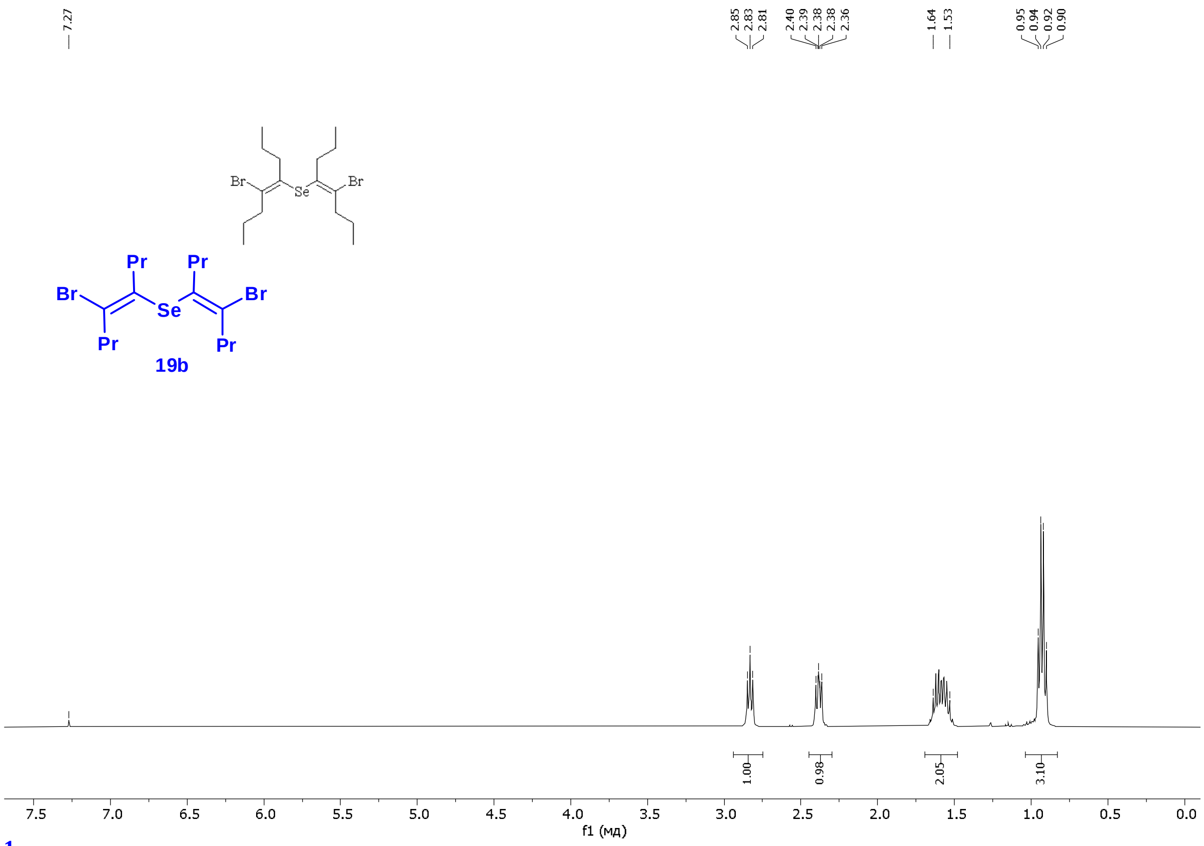

^{1}H-NMR spectrum of compound 19b

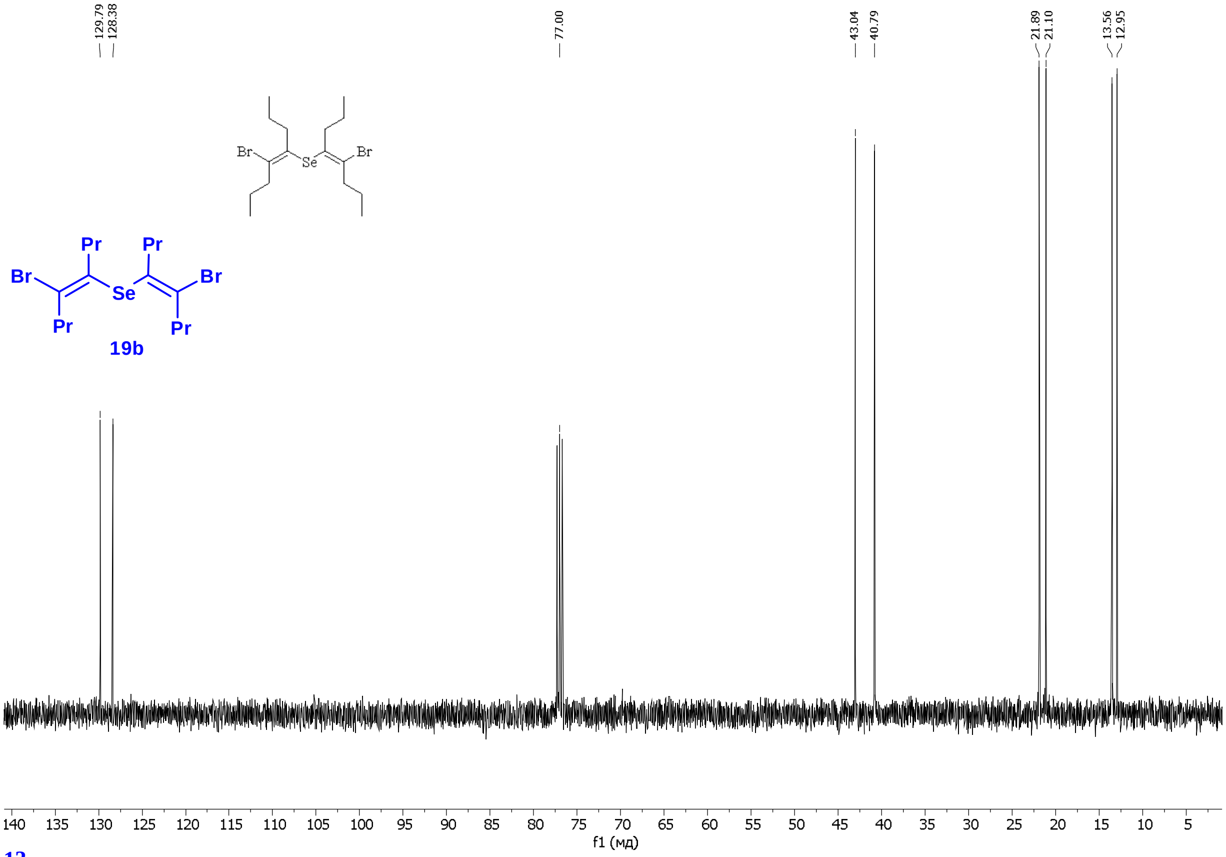

^{13}C-NMR spectrum of compound 19b

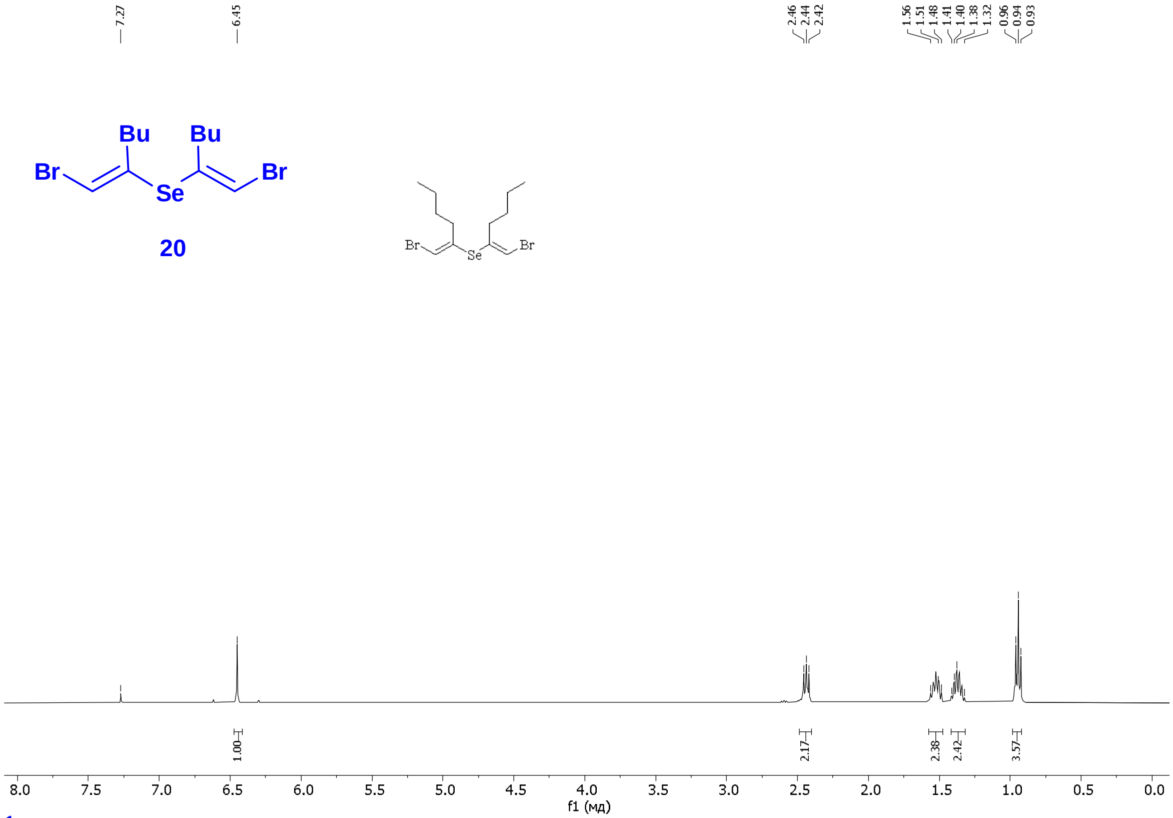

^{1}H-NMR spectrum of compound 20

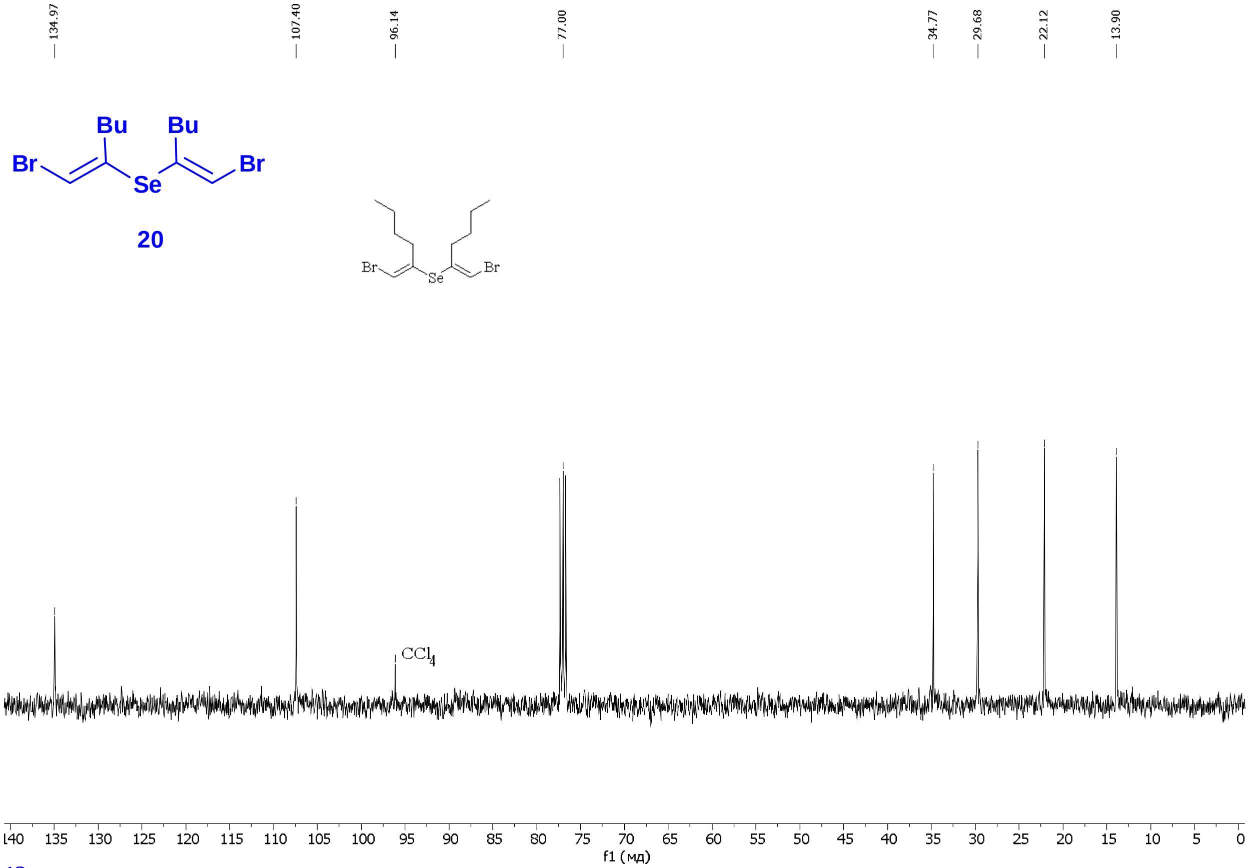

^{13}C-NMR spectrum of compound 20

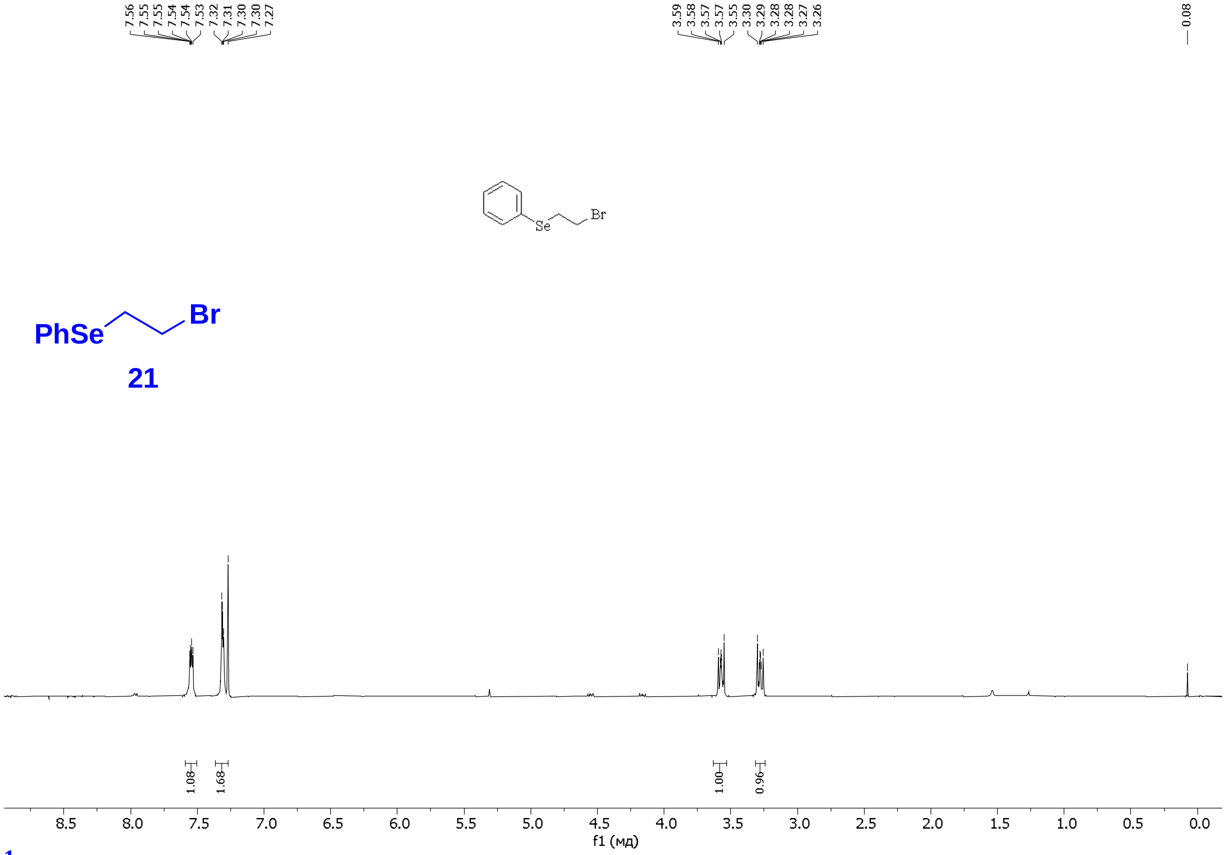

^{1}H-NMR spectrum of compound 21

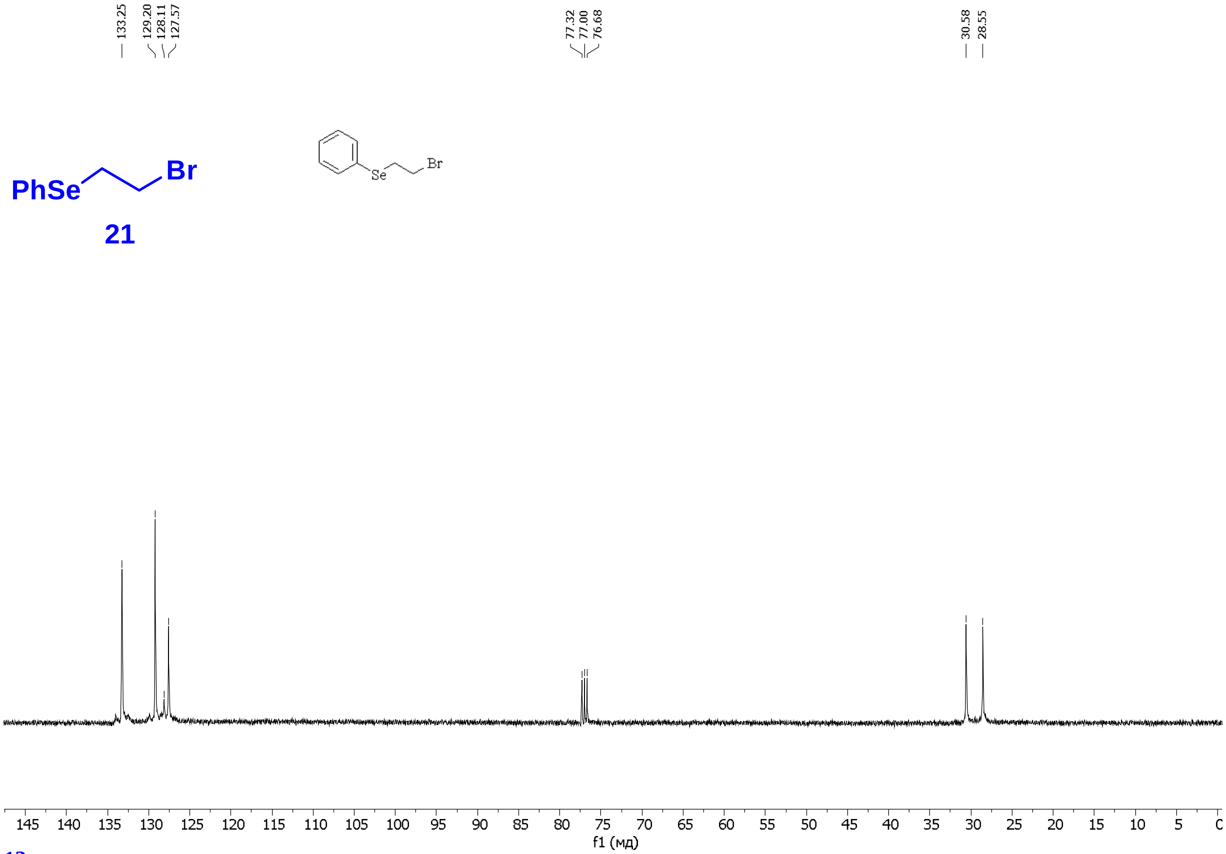

^{13}C-NMR spectrum of compound 21

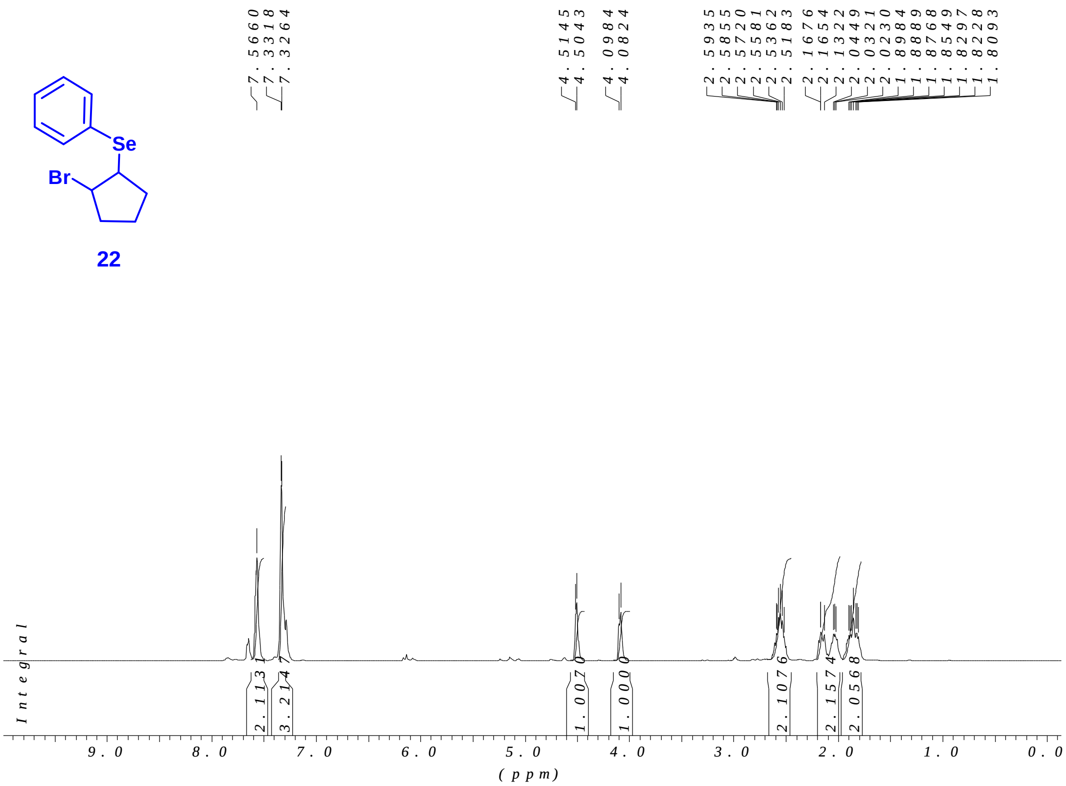

^{1}H-NMR spectrum of compound 22

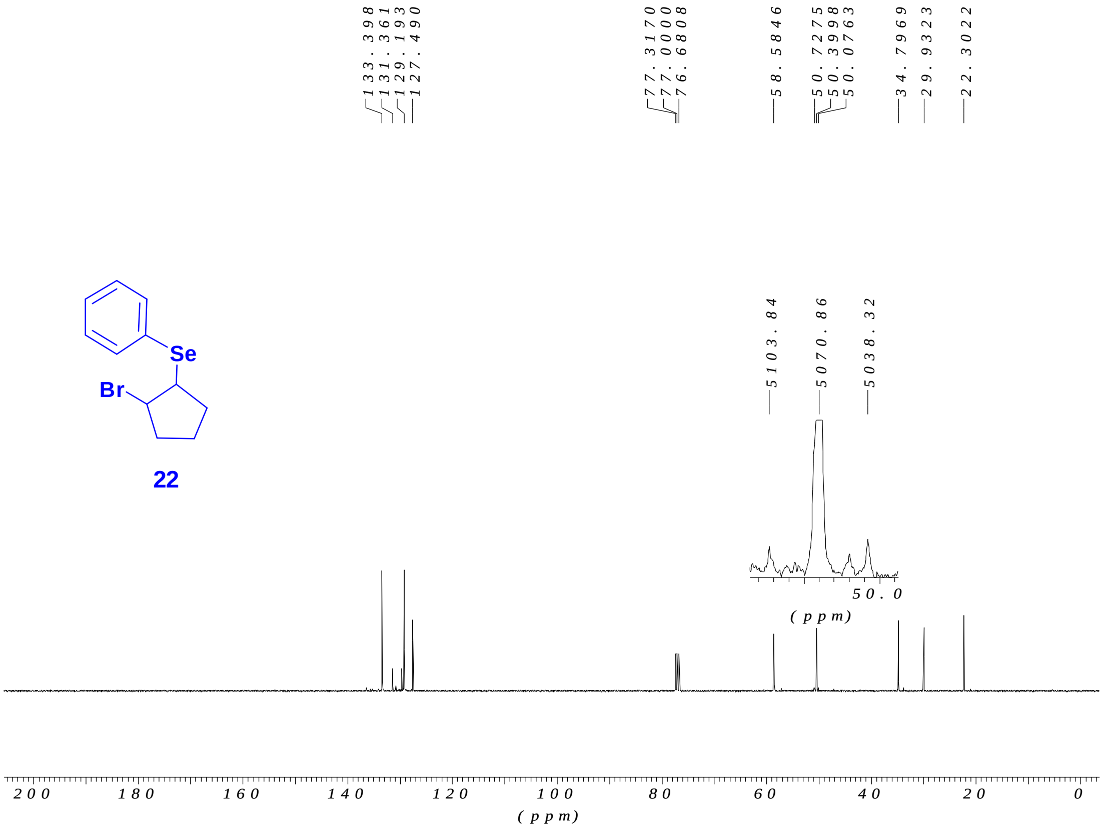

^{13}C-NMR spectrum of compound 22

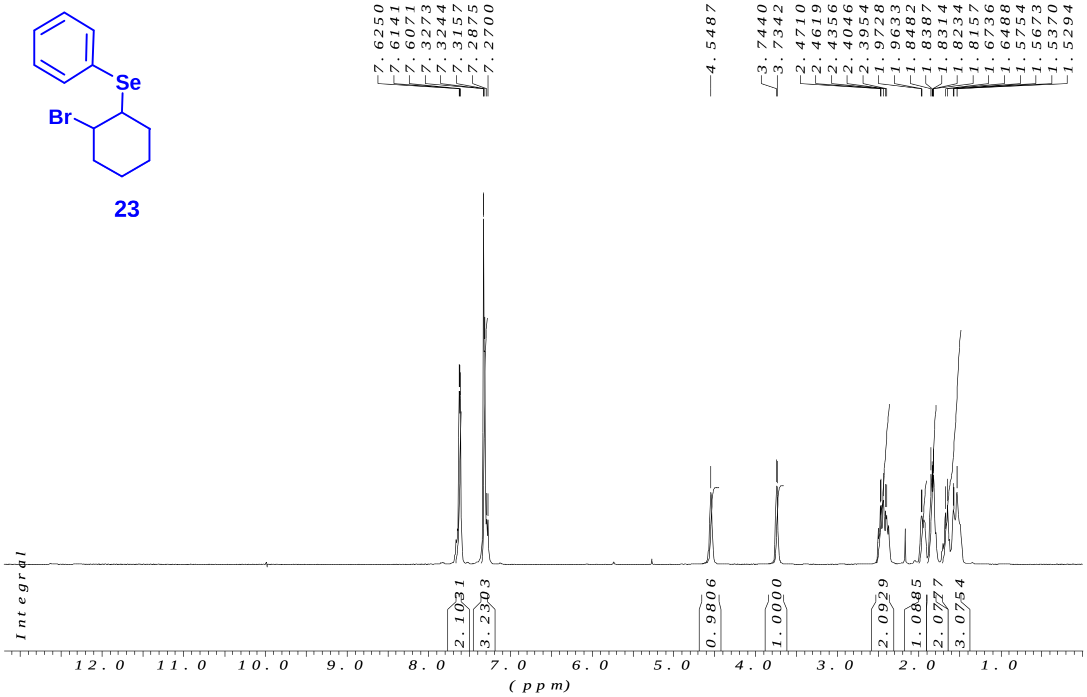

^{1}H-NMR spectrum of compound 23

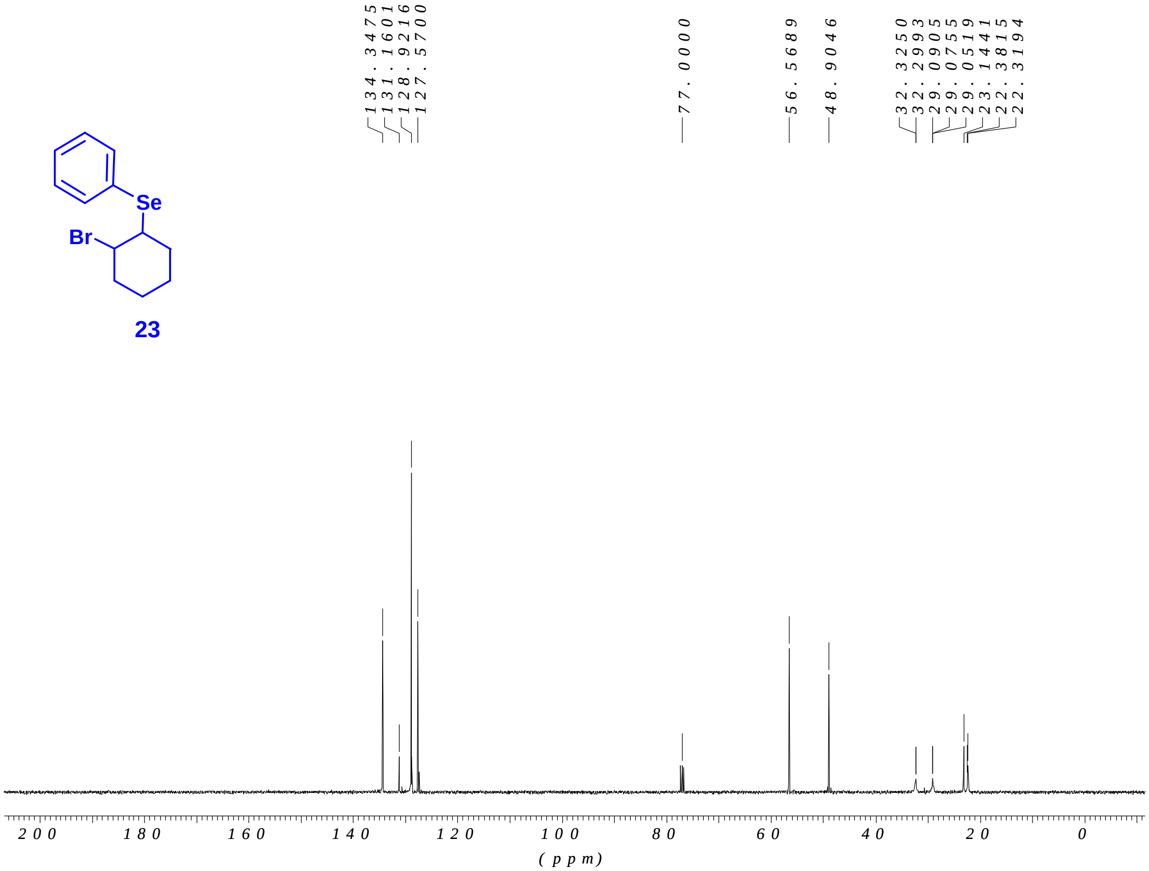

^{13}C-NMR spectrum of compound 23

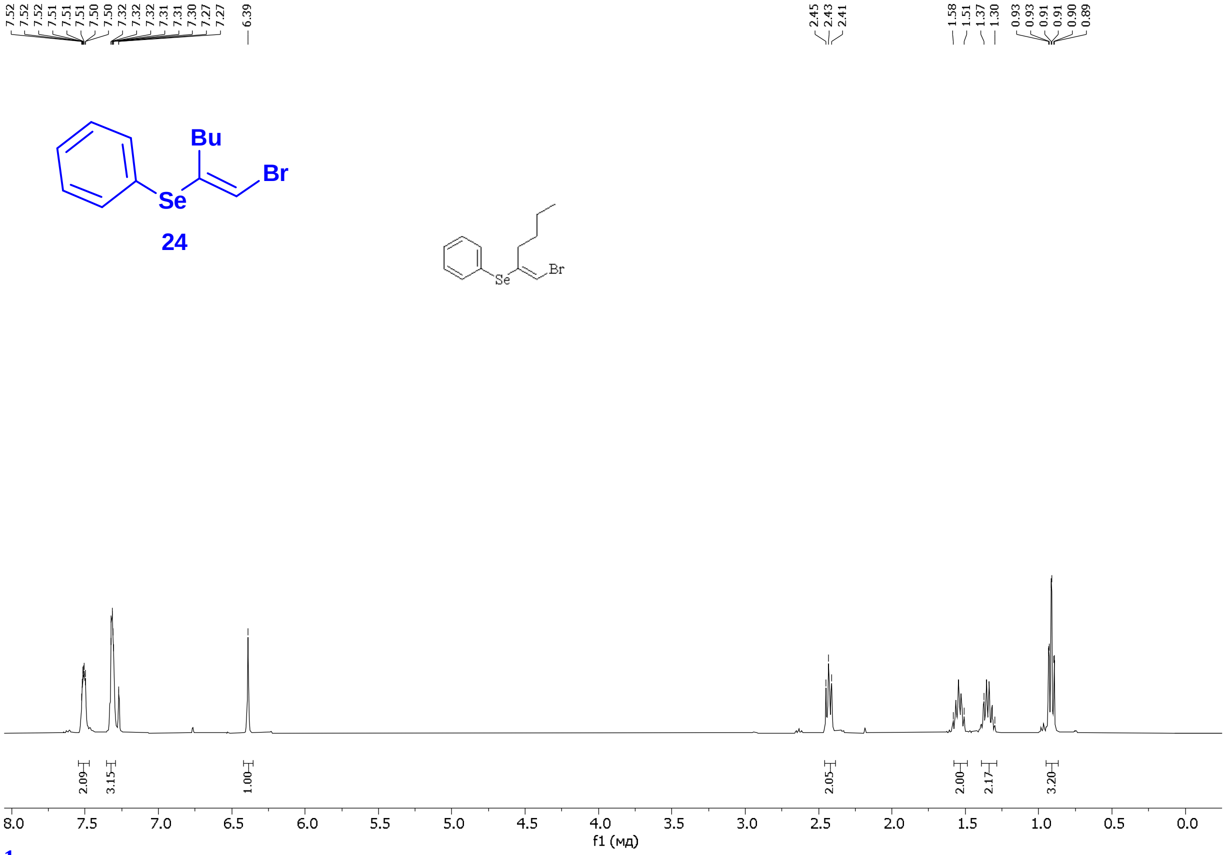

^{1}H-NMR spectrum of compound 24

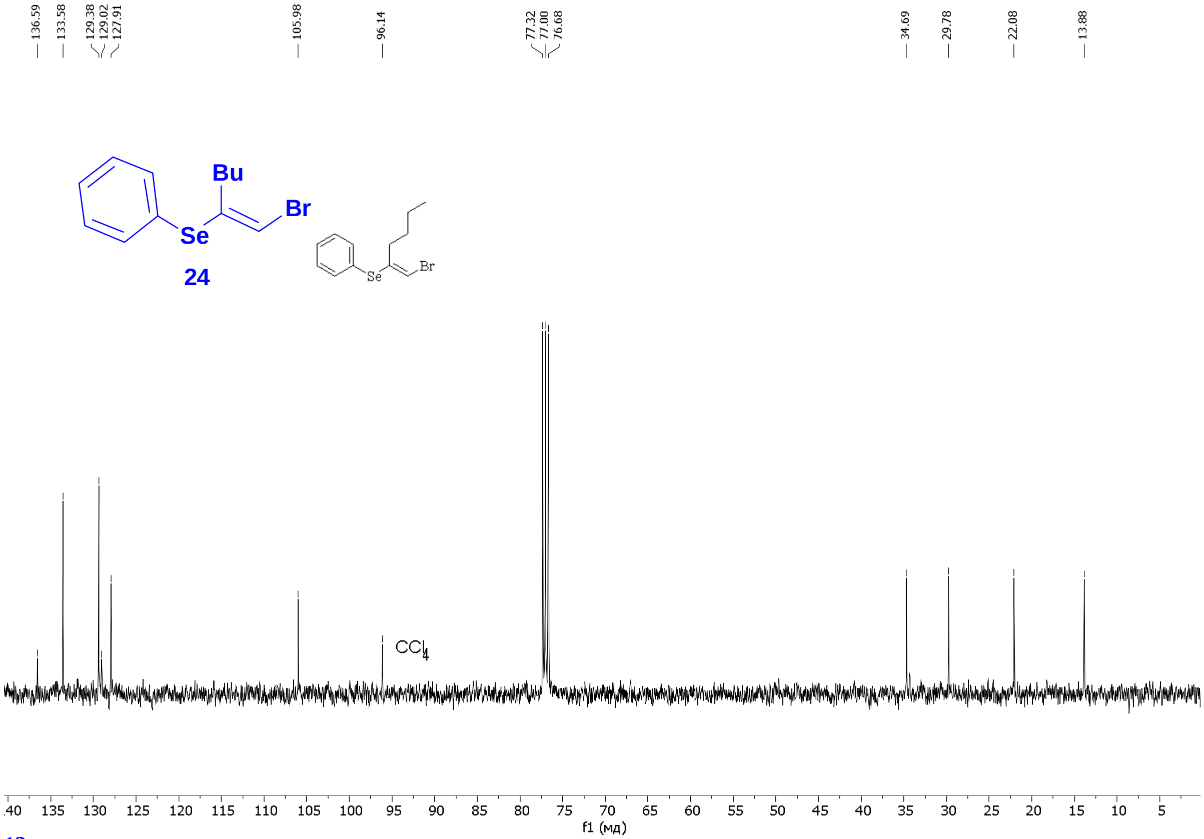

^{13}C-NMR spectrum of compound 24

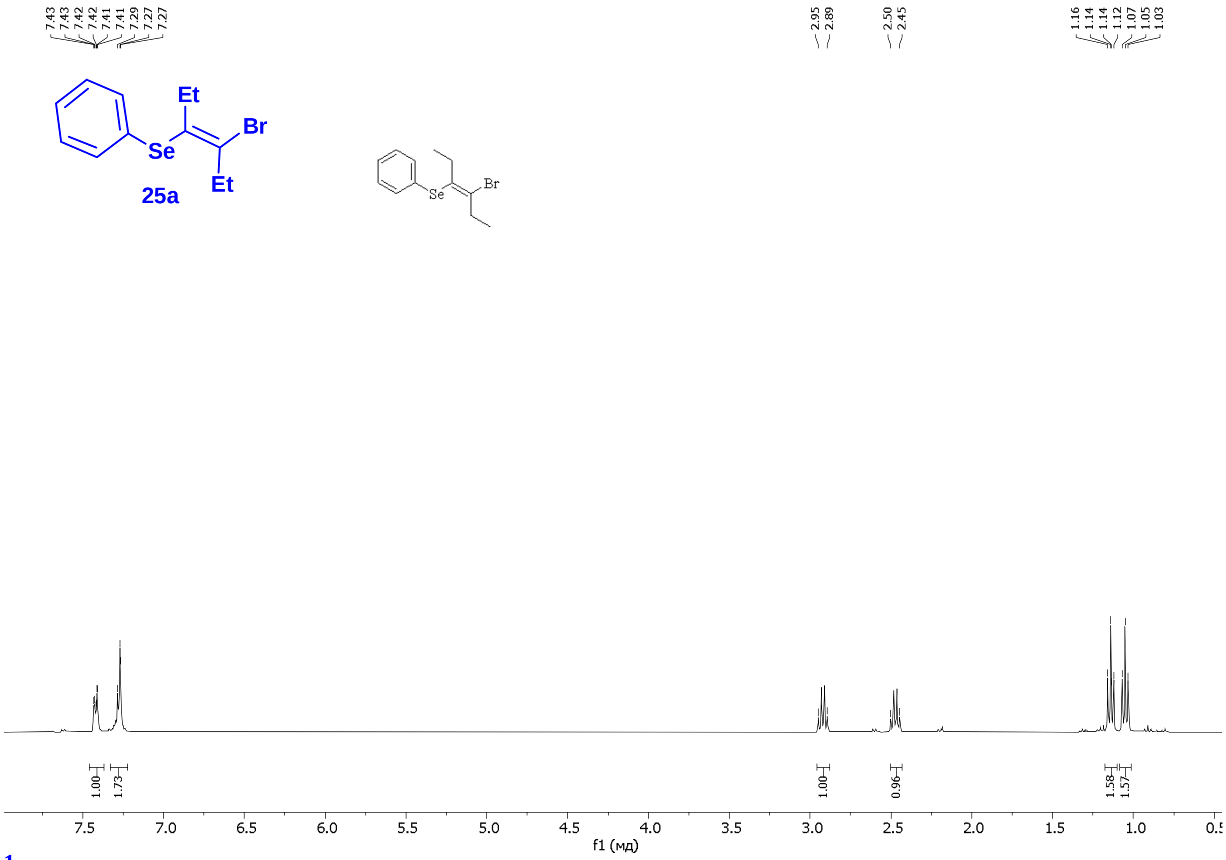

^{1}H-NMR spectrum of compound 25a

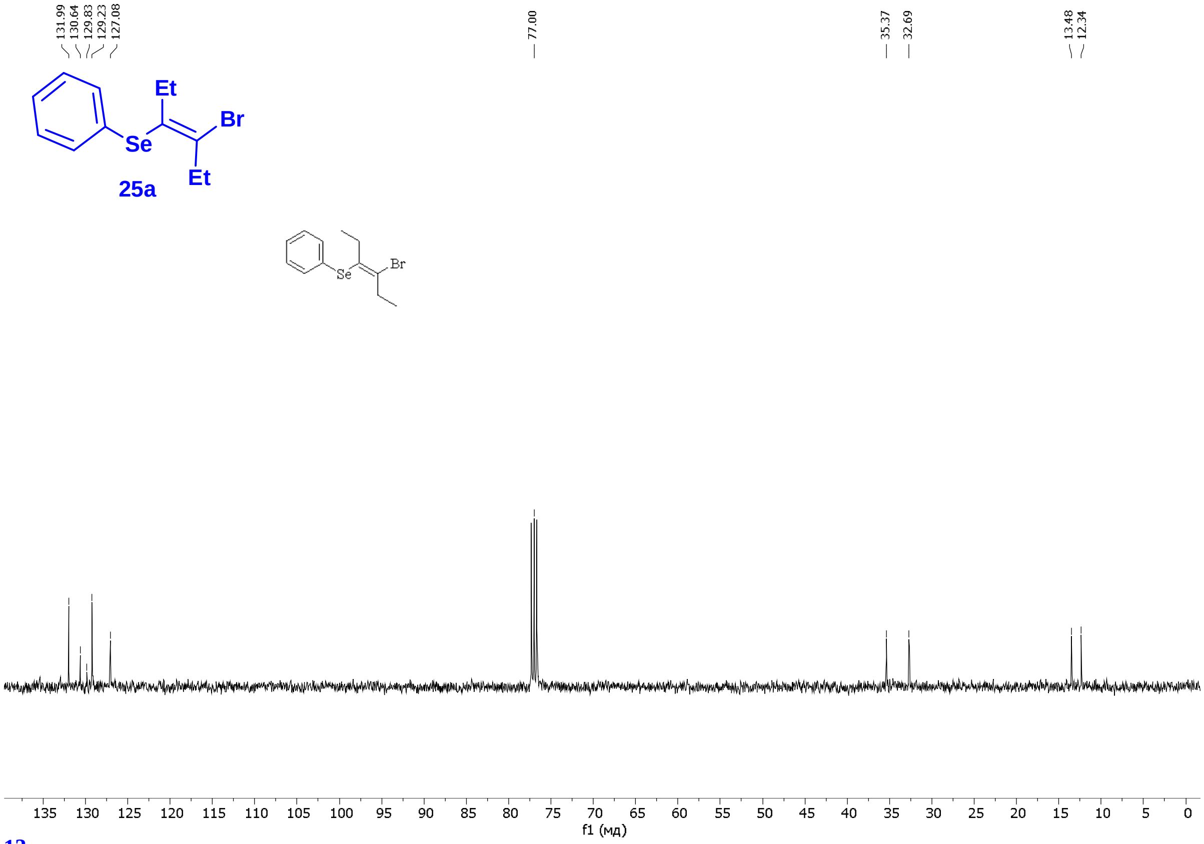

^{13}C-NMR spectrum of compound 25a

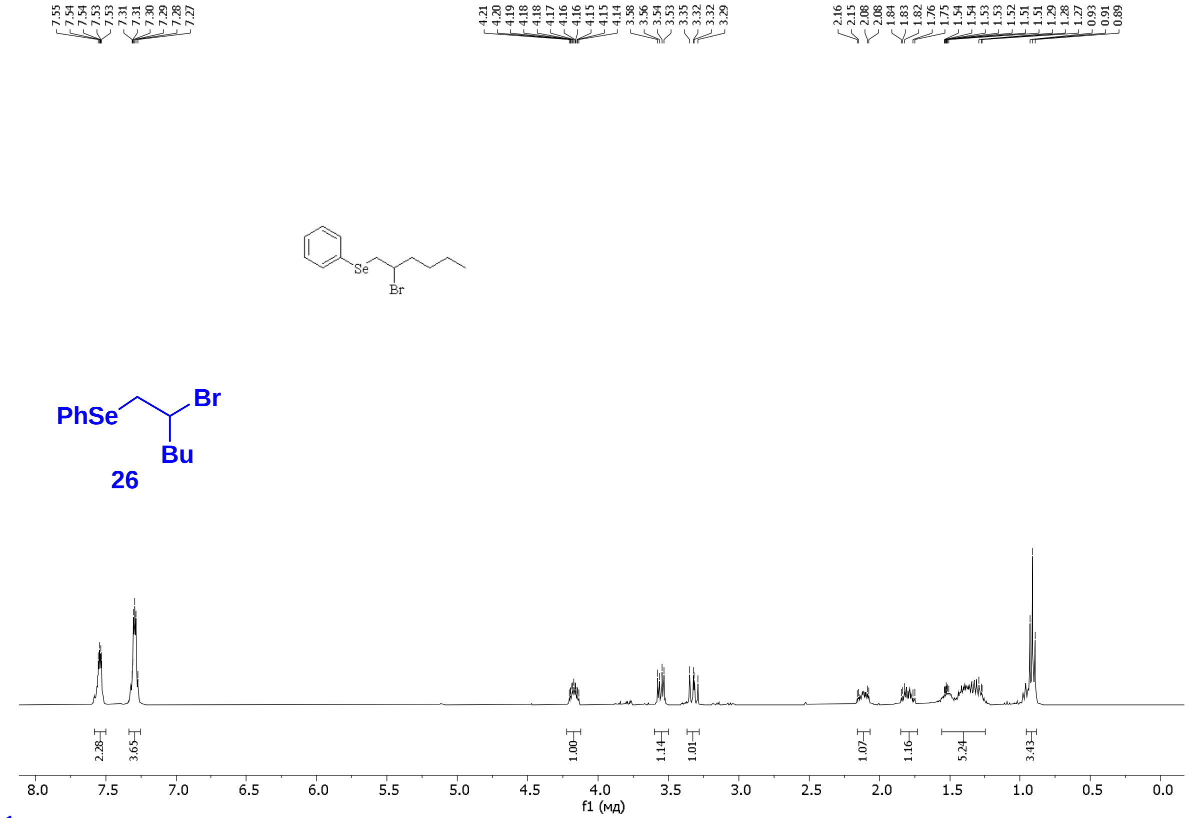

^{1}H-NMR spectrum of compound 26 (crude)

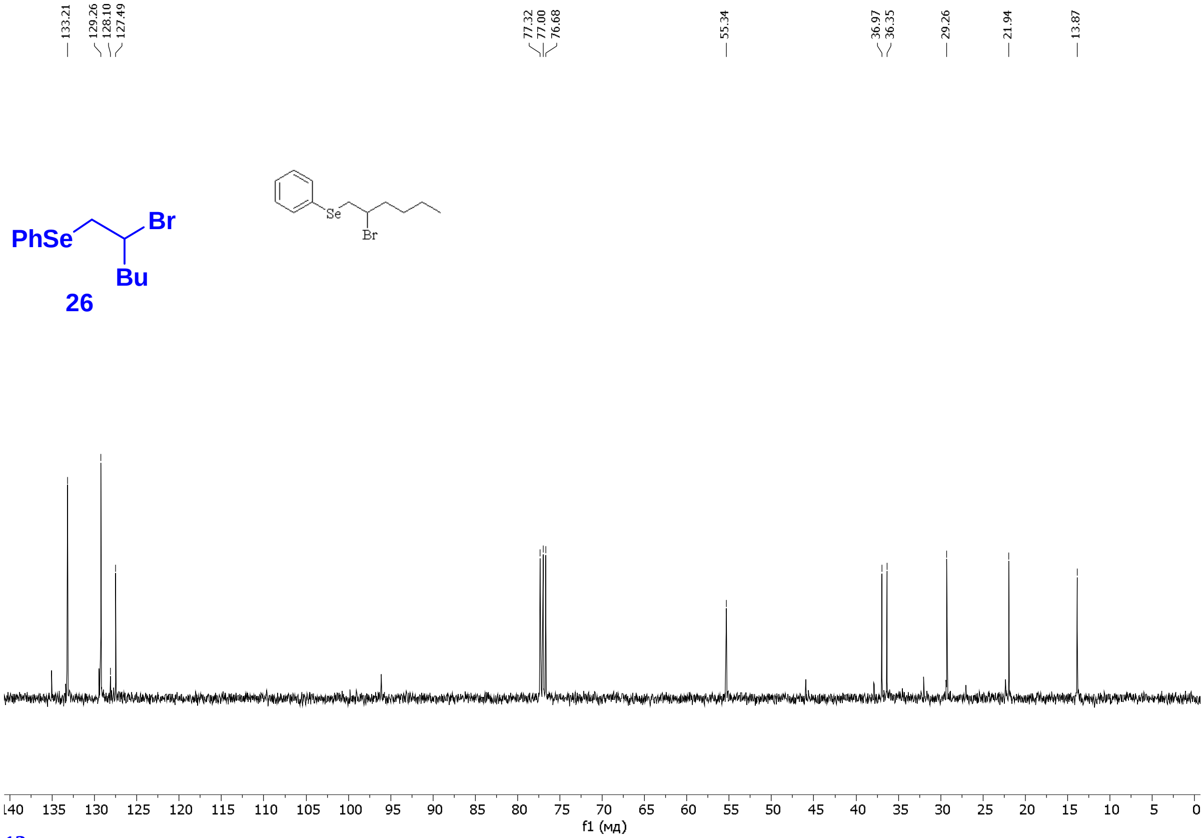

^{13}C-NMR spectrum of compound 26 (crude)

X-ray crystallographic study

Data were collected on a BRUKER D8 VENTURE PHOTON 100 CMOS diffractometer with MoK$_{\alpha}$ radiation (λ = 0.71073 Å) using the φ and ω scans technique. The structures were solved and refined by direct methods using the SHELX [1]. Data were corrected for absorption effects using the multi-scan method (SADABS). All non-hydrogen atoms were refined anisotropically using SHELX [1]. The coordinates of the hydrogen atoms were calculated from geometrical positions.

Crystal data and experimental details are given in Table 1.

Table 1 contains CCDC reference number of the supplementary crystallographic data for this paper. These data can be obtained free of charge from The Cambridge Crystallographic Data Centre via http://www.ccdc.cam.ac.uk

Table 1. X-ray crystallographic data for compounds **9a** and **10b**:

Compound	**9a**	**10a**
CCDC number	1965943	1502244
Empirical formula	$C_{10}H_{16}Cl_4Se$	$C_{10}H_{16}Br_4Se$
Formula mass [g/mol]	356.99	534.83
Temperature [K]	100(2)	100(2)
Crystalsistem	Monoclinic	Monoclinic
Space group	C2/c	C2/c
a [Å]	20.6655(16)	20.9331(9)
b [Å]	6.0655(5)	6.0827(3)
c [Å]	14.7555(19)	15.2530(11)
α [°]	90	90
β [°]	133.690(2)	132.9000(10)
γ [°]	90	90
V [Å^3]	1337.4(2)	1422.72(14)
Z	4	4
$D_{calcd.}$[g·cm^{-3}]	1.773	2.497

μ [mm^{-1}]	3.573	13.843
θrande[°]	2.3–27.00	2.66–30.06
Reflections collected	19559	22078
Independent reflections	1462 [R_{int}= 0.0638]	2090 [R_{int}= 0.0412]
R_1, wR_2 [I> 2σ(I)]	0.0266, 0.0633	0.0183, 0.0395
R_1, wR_2 (all data)	0.0274, 0.0637	0.0216, 0.0405
Completeness [%]	99.9	99.9
Crystal size [mm]	0.50×0.50×0.45	0.21×0.17×0.04
Goodness of fit	1.138	1.103

[1] Sheldrick G.M. // Acta Crystallogr. – 2008. – A64. – P. 112 – 122.
